# *Ppd-H1* integrates drought stress signals to control spike development and flowering time in barley

**DOI:** 10.1093/jxb/eraa261

**Published:** 2020-05-27

**Authors:** Leonard Gol, Einar B Haraldsson, Maria von Korff

**Affiliations:** 1 Institute for Plant Genetics, Heinrich-Heine University Düsseldorf, Düsseldorf, Germany; 2 Max-Planck-Institute for Plant Breeding Research, Cologne, Germany; 3 Cluster of Excellence on Plant Sciences, ‘SMART Plants for Tomorrows Needs’, Heinrich-Heine University Düsseldorf, Düsseldorf, Germany; 4 University of Glasgow, UK

**Keywords:** Barley, development, drought, flowering, *FLOWERING LOCUS T*, MADS-box genes, photoperiod, stress

## Abstract

Drought impairs growth and spike development, and is therefore a major cause of yield losses in the temperate cereals barley and wheat. Here, we show that the photoperiod response gene *PHOTOPERIOD-H1* (*Ppd-H1*) interacts with drought stress signals to modulate spike development. We tested the effects of a continuous mild and a transient severe drought stress on developmental timing and spike development in spring barley cultivars with a natural mutation in *ppd-H1* and derived introgression lines carrying the wild-type *Ppd-H1* allele from wild barley. Mild drought reduced the spikelet number and delayed floral development in spring cultivars but not in the introgression lines with a wild-type *Ppd-H1* allele. Similarly, drought-triggered reductions in plant height, and tiller and spike number were more pronounced in the parental lines compared with the introgression lines. Transient severe stress halted growth and floral development; upon rewatering, introgression lines, but not the spring cultivars, accelerated development so that control and stressed plants flowered almost simultaneously. These genetic differences in development were correlated with a differential down-regulation of the flowering promotors *FLOWERING LOCUS T1* and the BARLEY MADS*-*box genes *BM3* and *BM8.* Our findings therefore demonstrate that *Ppd-H1* affects developmental plasticity in response to drought in barley.

## Teaser

*Ppd-H1* integrates photoperiod and drought stress signals via *FLOWERING LOCUS T1* and the downstream MADS-box genes *BM3* and *BM8* to modulate reproductive development, and shoot and spike morphology in barley.

## Introduction

Global warming increases the frequency and intensity of severe water scarcity events, which negatively affect the yield of rain-fed crops such as barley (*Hordeum vulgare* L.) and wheat (*Triticum aestivum* L.) (Xie *et al.*, 2018; Kahiluoto *et al.*, 2019). Drought during reproductive development impairs spike development and floret fertility, and is therefore a major cause of yield losses in these temperate cereals (Gol *et al.*, 2017). At present, strategies to breed cereal varieties with improved yield under drought are limited due to a lack of knowledge of the genetic factors that control inflorescence and flower development under drought conditions. Understanding the plasticity and genetic control of stress-induced changes in reproductive development will be crucial to ensure future yield stability of temperate cereals.

The model plant *Arabidopsis thaliana* accelerates reproductive development under drought, a response that has been termed drought escape. In Arabidopsis, drought escape is triggered under inductive long-day (LD) conditions and is controlled by components of the circadian clock and the photoperiod response pathway (Riboni et al., 2013, 2016). Under drought conditions, the phytohormone abscisic acid (ABA) modulates the activity and signalling of the clock gene *GIGANTEA* (*GI*) and consequently its ability to activate *FLOWERING LOCUS T* (*FT*) under long photoperiods (Riboni et al., 2013, 2016). The FT protein acts as a florigenic signal, moving long distances from the leaf to the shoot apical meristem (SAM) to induce the floral transition (Abe *et al.*, 2005; Wigge *et al.*, 2005; Corbesier *et al.*, 2007; Jaeger and Wigge, 2007; Mathieu *et al.*, 2007; Tamaki *et al.*, 2007; Jaeger *et al.*, 2013). Under non-inductive short days (SDs), ABA delays flowering by repressing the flowering-promoting MADS-box gene *SUPPRESSOR OF OVEREXPRESSION OF CONSTANS1* (*SOC1*), encoding a transcription factor integrating floral cues in the shoot meristem (Riboni *et al.*, 2016). In addition, it was shown that ABA-responsive element (ABRE)-binding factors (ABFs) interact with NUCLEAR FACTOR Y subunit C (NF-YC) 3/4/9 to promote flowering by inducing *SOC1* transcription under drought conditions (Hwang *et al.*, 2019). On the other hand, ABSCISIC ACID-INSENSITIVE 3/4/5 bZIP transcription factors involved in ABA signalling repress flowering by up-regulating the floral repressor and vernalization gene *FLOWERING LOCUS C* (*FLC*) (Wang *et al.*, 2013; Shu *et al.*, 2016). Consequently, drought cues depend on the photoperiod and interact with photoperiod response and vernalization genes to modulate flowering time in Arabidopsis. In contrast to Arabidopsis, rice (*Oryza sativa* L.) shows a delay in flowering in response to drought under inductive photoperiods, and this delay is accompanied by a down-regulation of the florigenic signals *HEADING DATE 3a* (*Hd3a*) and *RICE FLOWERING LOCUS T 1* (*RFT1*) (Galbiati *et al.*, 2016; Zhang *et al.*, 2016). Consequently, the developmental response to drought varies within and between species, and is linked to the differential regulation of *FT*-like genes (Kazan and Lyons, 2016). However, the effects of drought on reproductive development and genetic components that modulate this response are not known in most crop species including the important temperate crop barley.

Barley germplasm is characterized by high genetic diversity and variation in response to abiotic stresses. While elite cultivars tend to be more stress susceptible, wild and landrace barley genotypes are well adapted to drought-prone environments and therefore represent a valuable resource for improving stress tolerance in elite barley (Baum *et al.*, 2007; von Korff *et al.*, 2008; Rollins *et al.*, 2013b; Templer *et al.*, 2017). It was demonstrated that yield stability in the field was associated with the major photoperiod response gene *PHOTOPERIOD H1* (*Ppd-H1*) and the vernalization gene *VERNALIZATION 1* (*VRN1*) (von Korff *et al.*, 2008; Rollins *et al.*, 2013a; Al-Ajlouni *et al.*, 2016; Wiegmann *et al.*, 2019). These findings suggested that the timing of reproductive development is crucial to maximize yield formation under harsh environmental conditions. However, it is not known if and how these floral regulators interact with stress cues to modulate development. *Ppd-H1*, a barley homologue of the PSEUDO RESPONSE REGULATOR (PRR) genes from the Arabidopsis circadian clock, induces the expression of *FLOWERING LOCUS T1* (*FT1*), a homologue of Arabidopsis *FT* and rice *Hd3a* under LDs (Turner *et al.*, 2005; Corbesier *et al.*, 2007; Tamaki *et al.*, 2007; Campoli et al., 2012*a*, *b*). In barley, the up-regulation of *FT1* in the leaf is correlated with induction of the MADS-box genes *VRN1* (*BM5a*), *BARLEY MADS-box 3* (*BM3*) and *BM8*, barley homologues of Arabidopsis *APETALA1/FRUITFUL* (*AP1/FUL*), and the acceleration of inflorescence development (Schmitz *et al.*, 2000; Trevaskis *et al.*, 2007; Digel *et al.*, 2015). Homologues of *Ppd-H1*/*PRR37* function in the circadian clock in Arabidopsis and rice (Makino *et al.*, 2001; Murakami *et al.*, 2003; Turner *et al.*, 2005). The circadian clock is an internal timekeeper that allows plants to anticipate predictable changes in the environment and controls a number of output traits including development and stress responses (Sanchez *et al.*, 2011; Müller *et al.*, 2014; Johansson and Staiger, 2015). In Arabidopsis, the central oscillator is composed of negative transcriptional feedback loops: the rise of *CIRCADIAN CLOCK ASSOCIATED1* (*CCA1*) and *LATE ELONGATED HYPOCOTYL* (*LHY*) late at night inhibits the evening complex genes *EARLY FLOWERING 3* (*ELF3*), *EARLY FLOWERING 4* (*ELF4*), and *LUX*, which in turn repress the *PRR* genes at night. Barley homologues of these clock genes have been identified and their interactions are largely conserved in barley (Campoli *et al.*, 2012b; Müller *et al.*, 2020). Accordingly, elements of the evening complex genes repress *Ppd-H1* at night and thereby control the photoperiod-dependent up-regulation of *FT1* (Faure *et al.*, 2012; Mizuno *et al.*, 2012; Zakhrabekova *et al.*, 2012; Campoli *et al.*, 2013; Alvarez *et al.*, 2016). In spring barley grown in northern latitudes, a recessive mutation in the CONSTANS, CONSTANS-like, and TOC1 (CCT) domain of *ppd-H1* has been selected (Jones *et al.*, 2008). This *ppd-H1* allele delays flowering under LDs and thereby improves yield in temperate environments with long growing seasons (Cockram *et al.*, 2007; Alqudah *et al.*, 2014; Digel *et al.*, 2015). In contrast, early flowering in response to LDs promoted by the wild-type *Ppd-H1* allele was associated with improved yield under Mediterranean environments with terminal stress (Wiegmann *et al.*, 2019). However, it is not known if the two *Ppd-H1* variants also interact with stress cues to modulate reproductive development.

Here, we provide a detailed analysis of barley development under drought. We show that variation at *Ppd-H1* interacts with drought to control flowering time, grain yield, as well as the expression of *FT1* and the downstream MADS-box genes *BM3* and *BM8*.

## Materials and methods

### Plant materials, growth conditions, and phenotyping

Drought responses were scored in the spring barley (*H. vulgare* L.) cultivars Scarlett, Golden Promise, and Bowman, and their derived introgression lines S42-IL107 (Scarlett), GP-fast (Golden Promise), and BW281 (Bowman). Scarlett, Golden Promise, and Bowman carry a natural mutation in the CCT domain of *Ppd-H1*, that causes a delay in flowering under LD conditions (Turner *et al.*, 2005). The derived introgression lines S42-IL107 and BW281 carry a dominant *Ppd-H1* allele introgressed from wild and winter barley, respectively (Druka *et al.*, 2011; Schmalenbach *et al.*, 2011). GP-fast was created via crossing of Golden Promise to the winter barley cultivar Igri, followed by two rounds of backcrossing to Golden Promise to reduce the size of the introgression.

The three spring barley cultivars and derived introgression lines were genotyped with the Barley 50k iSelect SNP Array at TraitGenetics GmbH (Gatersleben, Germany) (Bayer *et al.*, 2017). Chromosomal positions for each marker were obtained from the POPSEQ_2017 genetic map (Cantalapiedra *et al.*, 2015; Mascher *et al.*, 2017). Sizes of the introgressions were calculated based on half the distance between the markers flanking donor introgressions and the first polymorphic markers within the introgressions (Supplementary Fig. S1; Supplementary Data S1 at *JXB* online).

We conducted two different drought experiments. First, a continuous drought treatment was applied by a controlled dry down of the soil to a soil water content (SWC) of 15% of field capacity (FC), and this FC was maintained until plant maturity. In a second experiment, a transient drought treatment was applied by withholding water for eight consecutive days during floral development followed by rewatering to control levels. Both experiments were performed in a controlled-environment chamber under 60% relative humidity. Individual grains were sown in 7 cm×7 cm×8 cm black plastic pots; 40 pots (5×8 rows) per tray. Genotypes were distributed randomly on each tray and rearranged after each sampling to maintain the initial planting density. Additionally, trays were rotated and shuffled at least twice per week. Each pot was filled with exactly 150 g of soil mixture. A mixture of 93% (v/v) Einheitserde ED73 (Einheitserde Werkverband e.V., Sinntal_Altengronau, Germany), 6.6% (v/v) sand, and 0.4% (v/v) Osmocote exact standard 3–4M (Scotts Company LLC), was freshly prepared before sowing. This porous soil mixture with high organic matter content was selected to further aid the even distribution of moisture in the soil. Grains were stratified in well-watered soil at 4 °C in the dark for at least 4 d. Plants were then germinated under SD conditions (8 h, 22 °C day; 16 h, 18 °C night; photosynthetically active radiation ~250 µM m^–2^ s^–1^). For the continuous drought treatment, water was withheld after germination until the SWC reached 15% FC, while the control plants were watered to maintain 70% FC. The desired SWC of 15% FC was reached after 10 d when all plants were transferred from SDs to LDs and kept under LDs for the rest of the experiment (16 h, 22 °C day; 8 h, 18 °C night; photosynthetically active radiation ~250 µM m^–2^ s^–1^). For the application of severe transient drought, plants of Scarlett and S42-IL107 were germinated under SD conditions and shifted to LDs after 10 d. All plants were kept at 70% FC until they had reached the awn primordium stage [Waddington stage 3 (W3)]. Then watering was stopped for eight consecutive days. SWC in the pots reached a relative water content (RWC) of 8% FC on the eighth day. Control plants were kept at 70% FC during this time. Subsequently, all drought-treated pots were rewatered to control levels of 70% FC. FC was calculated from the difference in weight of fully hydrated and oven-dried soil. SWC was measured gravimetrically (Coleman, 1947). Pots were soaked with water and subsequently left to drain by gravity until their weight remained stable; this was set as 100% FC. Dry weight was measured after pots were dried in a drying cabinet at ~60°C until their weight remained stable. Measurements of FC were corrected for the biomass accumulation of growing plants as the experiments progressed by subtracting the weight of harvested plants from the measured soil weight. The weight of pots was checked daily and all plants were watered daily to maintain the same SWC throughout development. At least three replicate plants of all six genotypes were sown and germinated for each sampling time point.

The development of the main shoot apex (MSA) was scored in accordance with the stages described by Waddington *et al.* (1983) that is based on the progression of inflorescence initiation and then the most advanced floret primordium and pistil of the inflorescence. At W2 the first spikelets initiate and the MSA transitions to a reproductive inflorescence. The first floral organ primordia differentiate and stem elongation initiates at the stamen primordium stage (W3.5). New spikelet primordia are continuously initiated until about W5, which then mature into florets until anthesis and pollination at W10. MSA dissection was performed with microsurgical stab knives (SSC#72-1551; Sharpoint, Surgical Specialties Corporation). Images of developing apices were obtained using a Nikon stereo microscope (Nikon SMZ18), Nikon DS-U3 controller unit, and a Nikon DS-Fi2 digital camera. Nikon NIS-Elements software was used for image acquisition. Heading date was scored at Zadoks stage Z49 when first awns became visible, otherwise also referred to as tipping (Zadoks *et al.*, 1974; Alqudah and Schnurbusch, 2017). Spike number, the number of grains per spike, the number of grains per plant, and thousand kernel weight (TKW) were scored at harvest.

Leaf RWC was determined from measurements of fresh, turgid, and dry weight of leaf sections from the middle part of the youngest fully expanded leaf. Turgid weight was measured after soaking the leaf sections in deionized water at 4 °C overnight in the dark. Dry weight of leaf sections was measured after drying at 70°C. The RWC was then calculated as (Smart and Bingham, 1974).

### RNA extraction and gene expression analysis

Sections from the middle of the youngest fully emerged leaf were sampled for the developmental time courses at Zeitgeber time 8 (ZT8). Sampling was started on the first day after transfer to LDs in the continuous drought treatment and as soon as water was withheld in the severe drought experiment. Sampling was continued until flowering for both treatments. Samples for the diurnal expression analyses were harvested every 4 h starting at ZT0, with one additional sampling at ZT22. RNA extraction, reverse transcription, and quantitative real-time PCR (qRT-PCR) were performed as previously described (Campoli et al., 2012*a*, *b*; Digel *et al.*, 2015). Several combinations of reference genes were tested for each experiment, and the genes with the most stable expression were chosen for normalization. The geometric mean of *Actin* and *ADP-ribosylation factor 1-like protein* (*ADP*) absolute expression was used for the calculation of relative gene expression levels for the developmental time courses. The geometric mean of *ADP* and *Glyceraldehyde-3-phosphate dehydrogenase* (*GAPDH*) absolute expression was used for the calculation of relative gene expression levels for the diurnal time course. Normalization was performed by dividing target gene expression values by the obtained mean of the reference genes.

### Statistical analysis

All statistical analyses were performed with R (R Core Team, 2020). Polynomial regressions (Loess smooth line) were calculated using second-degree polynomials and an alpha of 0.75, with a 95% confidence interval. Student’s *t*-test assuming two-tailed distribution and equal variance was used to compare group means for control and drought treatments at each time point of the time course analyses with a significance cut-off of *P*<0.05. Significant differences in trait expression between treatments and genotypes were compared by Kruskal–Wallis ANOVA followed by Conover–Iman test for multiple comparisons and Bonferroni correction with a significance cut-off of *P*<0.05.

## Results

### Drought interacts with *Ppd-H1* to modulate flowering time

We aimed to characterize the effects of drought on the timing of reproductive development and on shoot and spike morphology. In addition, we tested if the major photoperiod response gene *Ppd-H1* controlled reproductive development in response to drought. We quantified the effects of drought on developmental timing, growth, and inflorescence morphology in the spring barley genotypes Scarlett, Golden Promise, and Bowman with a natural mutation in the CCT domain of *Ppd-H1* and in the derived introgression lines S42-IL107 (Scarlett), GP-fast (Golden Promise), and BW281 (Bowman) that carry wild-type *Ppd-H1* alleles introgressed from wild barley (*H. vulgare* ssp*. spontaneum*) or winter barley (Supplementary Fig. S1) (Druka *et al.*, 2011; Schmalenbach *et al.*, 2011).

**Fig. 1.**
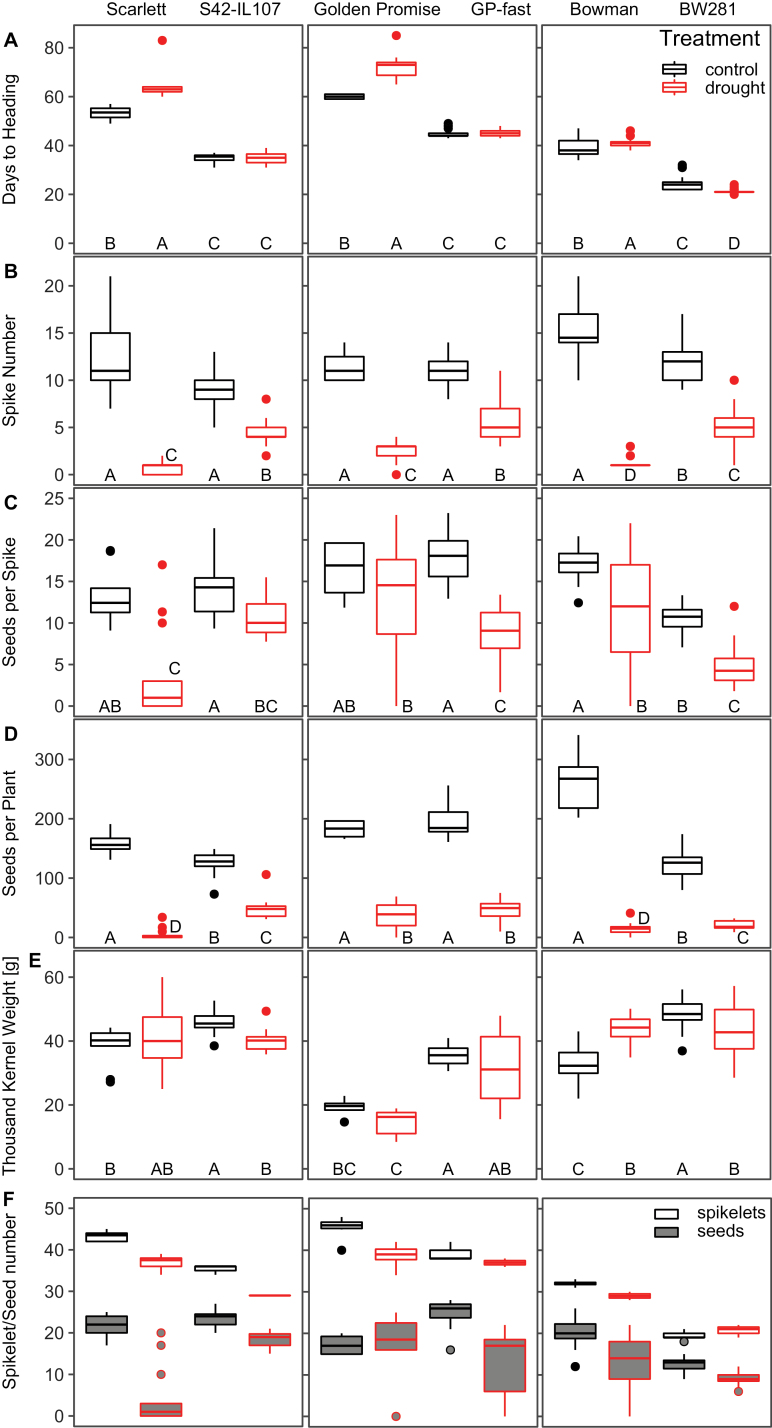
Continuous drought affects heading date, and shoot and spike morphology in barley. Days to heading (A), spike number per plant (B), grain number per spike (C), the number of grains per plant (D), thousand kernel weight (TKW) (E), and the maximum number of developed spikelets (unfilled boxes) and the number of grains (grey box) (F) were scored under control (black) and drought (red) conditions under LDs (16 h light/8 h night) in the spring barley cultivars Scarlett, Golden Promise, and Bowman, and the derived introgression lines S42-IL107, GP-fast, and BW281. Statistical groups were assigned using Kruskal–Wallis ANOVA and post-hoc Conover–Iman test and Bonferroni correction. Different letters indicate that groups differ (*P*<0.05).

We developed an assay to apply drought starting from early vegetative growth and lasting until maturity. With this assay, drought effects on the transition of vegetative to reproductive development and on floral progression were examined. Heading date, scored as a proxy for flowering time, was significantly delayed in all parental spring barley genotypes (Supplementary Fig. S2). Heading date was delayed by 11 d in Scarlett, by 13 d in Golden Promise, and by 3 d in Bowman under drought compared with control conditions (Fig. 1A). In contrast, heading date was not significantly different under drought compared with control conditions in S42-IL107 and GP-fast, and was significantly accelerated in BW281. In the parental genotypes, the number of spikes per plant was strongly reduced under drought; all plants produced only a maximum of three spikes under drought compared with >10 spikes under control conditions (Fig. 1B). The introgression lines produced on average 5–6 spikes per plant under drought compared with twice as many under control conditions, and thus significantly more under drought compared with the parental genotypes. Drought also reduced the number of grains per spike in all genotypes (Fig. 1C). However, there were no consistent differences in the reduction of grain number between *Ppd-H1* variants. The reductions in the number of spikes per plant and grains per spike resulted in a severely reduced number of grains per plant under drought (Fig. 1D). Total grain numbers under drought were significantly higher in the introgression lines S42-IL107 and BW281 than in the parental lines and not significantly different between Golden Promise and GP-fast. Drought did not strongly influence the TKW. Total yield per genotype was therefore primarily determined by the grain number (Fig. 1E).

We further investigated at which stage drought reduced final grain number and evaluated the effects of drought on spikelet versus grain number. Drought reduced the number of initiated spikelets in Scarlett, S42-IL107, Golden Promise, and Bowman by between 9% in Bowman to 18% in S42-IL107, while spikelet numbers were not significantly different between control and drought in BW281 and GP-fast (Fig. 1F). Furthermore, not all spikelets on the main spike developed grains. Under control conditions, the number of grains compared with initiated spikelets was reduced by 34–37% in the introgression lines and by 37% in Bowman, 50% in Scarlett, and 62% in Golden Promise. Consequently, in S42-IL107, GP-fast, and BW281, a higher percentage of spikelets developed grains compared with Scarlett, Golden Promise, and Bowman, respectively. Under drought conditions, the number of grains per spikelet was even more strongly reduced in all genotypes compared with control conditions, except for Golden Promise and S42-IL107. Under drought, relative grain numbers compared with spikelet numbers were reduced by 88% in Scarlett, by 64% in GP-fast, and by 56% and 57% in Bowman and BW281, respectively. Consequently, the reduction in grain number per spike under drought was primarily caused by an abortion of florets or floret sterility rather than a decrease in spikelet numbers.

**Fig. 2.**
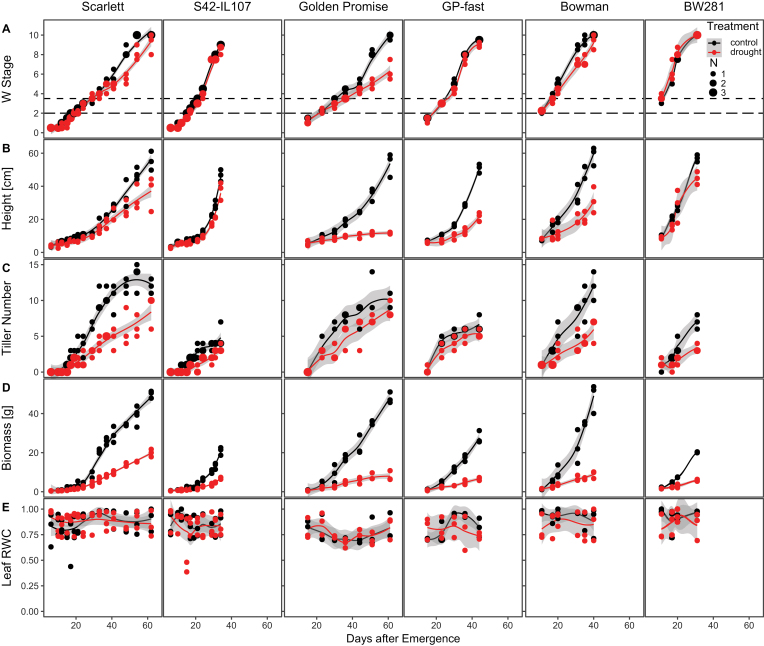
Continuous drought delays floral development in spring barley. Development of the main shoot apex (MSA) (A), plant height (B), the number of tillers (C), fresh weight biomass (D), and leaf relative water content (RWC) (E) were scored during development under control (black) and drought (red) conditions under LDs (16 h light/8 h night) in the spring barley cultivars Scarlett, Golden Promise, and Bowman, and their derived introgression lines S42-IL107, GP-fast, and BW281 according to the scale by Waddington *et al.* (1983). Dot sizes indicate the number of overlapping samples. Trend lines were calculated using a polynomial regression (Loess smooth line); grey areas show the 95% confidence interval.

Development of the MSA was scored after microdissection according to the scale established by Waddington *et al.* (1983) (Supplementary Fig. S3). The timing of spikelet initiation was not significantly altered by drought in any of the genotypes (Fig. 2A). However, drought delayed floral progression in the parental genotypes, but not in the introgression lines. Similarly, stem elongation, measured as plant height, was strongly reduced under drought in the three parental genotypes, but was less affected in the introgression lines (Fig. 2B). Variation at *Ppd-H1* and drought also had strong effects on the progression of tiller development (Fig. 2C). The introgression lines developed significantly fewer tillers than the parental lines under control and drought conditions. Drought delayed the development of tillers in Scarlett, Bowman, and BW281, but tiller development was not significantly different in S42-IL107, Golden Promise, and GP-fast. Consequently, drought had a much stronger effect on spike number than tiller number, demonstrating that the plants produced tillers during drought that did not develop a spike (Fig. 1B). The faster reproductive development in the introgression lines correlated with a reduced biomass accumulation compared with the parental lines under control and drought conditions. Drought reduced fresh weight biomass in all lines, and the relative reductions were similar between the parental genotypes and their respective introgression line. For example, 34 d after emergence, an ~70% reduction in biomass was observed in both Scarlett and S42-IL107 (Fig. 2D). We did not observe any effect of drought on the phyllochron and the number of leaves on the main culm, but leaf size was strongly reduced under drought (Supplementary Fig. S4). Leaf RWC was not altered under drought in any of the tested lines, indicating that all plants responded to the reduced water availability through a growth reduction and thus avoided tissue dehydration (Fig. 2E).

**Fig. 3.**
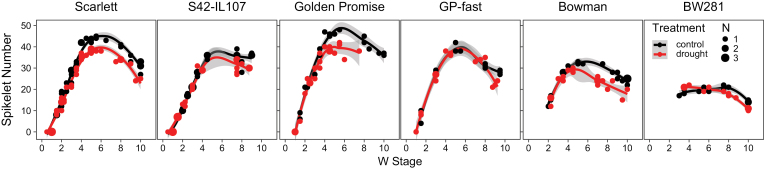
Continuous drought affects tillering and spikelet number in barley. The number of spikelets on the main shoot apex (MSA) (A) were scored during development under control (black) and drought (red) conditions under LDs (16 h light/8 h night) in the spring barley cultivars Scarlett, Golden Promise, and Bowman, and the derived introgression lines S42-IL107, GP-fast, and BW281. Dot sizes indicate the number of overlapping samples. Trend lines were calculated using a polynomial regression (Loess smooth line); grey areas show the 95% confidence interval.

The induction of spikelets on the MSA terminated earlier in the introgression lines which therefore formed fewer spikelets compared with their respective parents. The introgression lines initiated spikelets until W4–5 while the parental lines formed new spikelets until W5–6 (Fig. 3). Under drought, the initiation of spikelets was slowed down in the parental lines, so that fewer spikelets were initiated under drought than under control conditions. However, in the introgression lines, there was no significant difference in the initiation of spikelet primordia between control and drought conditions. While the parental lines initiated more spikelets than the introgression lines, a higher proportion of spikelets did not develop florets in the parental genotypes, compared with the introgression lines. The introgression lines initiated fewer spikelets under control conditions, but drought did not reduce spikelet number further in these lines. The differences between spikelet number and grain number observed in the introgression lines (Fig. 1F) were therefore due to low floret fertility and not a failure in developing florets.

Taken together, *Ppd-H1* controlled the drought-induced changes in reproductive development, shoot and spike morphology, and plant height. Elite spring barley with a mutation in *ppd-H1* displayed a strong delay in floral development and reductions in plant height and the number of spikelets initiated on the main inflorescence under drought, whereas these traits were scarcely affected under drought in the introgression lines with a wild-type *Ppd-H1* allele. Finally, drought had a strong detrimental effect on floret fertility which resulted in a reduction of grains independent of the *Ppd-H1* genotype.

### *Ppd-H1* affects the plasticity of reproductive development in response to a transient drought stress

**Fig. 4.**
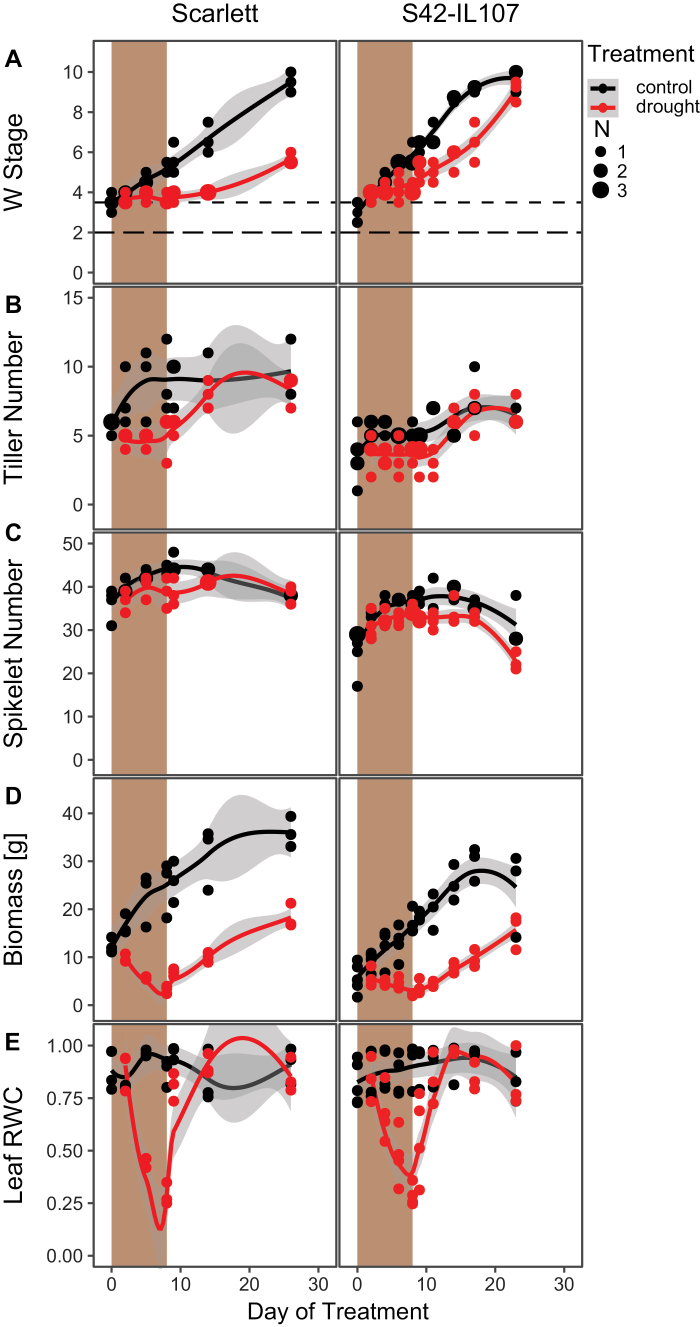
Severe drought delays MSA development in barley. MSA development (A), the number of tillers (B), the number of spikelets (C), fresh weight biomass (D), and leaf RWC (E) were scored under control (black) and severe drought (red) conditions and during recovery. Shaded areas indicate the period during which plants were not watered. Dot sizes indicate the number of overlapping samples. Trend lines were calculated using a polynomial regression (Loess smooth line), grey areas show the 95% confidence interval.

The severity, duration, and timing of drought events are highly variable in nature. We therefore tested if the observed effects of drought on reproductive development are dependent on the timing and severity of the stress. In addition, we investigated if *Ppd-H1* also affected the plasticity of development in response to a transient drought stress followed by a recovery phase. Under severe drought, reproductive development stopped completely in Scarlett for the duration of the stress treatment and resumed after rewatering (Fig. 4A). However, the delay in development was maintained after the stress treatment, and stressed plants flowered significantly later than control plants. In S42-IL107, reproductive development only slowed down after the onset of drought stress and did not stop completely. After rewatering, reproductive development even accelerated so that control and stressed plants flowered almost at the same time (Fig. 4A; Supplementary Fig. S5). Tiller development was also halted in both genotypes upon the onset of stress, but both genotypes resumed tiller development after rewatering, and tiller numbers were not significantly different between control and stress conditions at flowering. Spikelet numbers were not strongly altered during development because at the onset of drought (W3) the majority of spikelets had already initiated. Drought, however, still caused a small reduction in spikelet initiation in both genotypes. The treatment completely stopped biomass accumulation in both genotypes already after 2 d of withholding water. On the eighth day, when the drought level was most severe, control plants of both Scarlett and S42-IL107 had accumulated almost nine times as much biomass compared with drought-stressed samples. The reductions in fresh biomass were also caused by a strong decline in the leaf RWC upon application of the severe drought stress (Fig. 4D, E). However, after rewatering, RWC levels rapidly increased again and were similar to RWC levels in control plants 6 d after rewatering in both genotypes. While RWC levels fully recovered after rewatering and stressed plants resumed growth, fresh weight biomass was significantly lower in stressed compared with control plants at flowering.

Taken together, transient severe stress applied during stem elongation also delayed floral development as observed under mild stress. Interestingly, the introgression line but not the parental line accelerated reproductive development after rewatering. Stressed and control S42-IL107 plants flowered nearly simultaneously, suggesting that *Ppd-H1* affects the developmental plasticity in response to drought.

### Drought alters the expression of clock and floral regulator genes in barley

Components of the circadian clock play important roles in the control of flowering time regulators in barley. Additionally, previous studies have found that abiotic stresses alter the diurnal gene expression of core clock genes and clock-regulated genes in barley (Habte *et al.*, 2014; Ford *et al.*, 2016; Ejaz and von Korff, 2017). We therefore examined whether reduced SWC affected reproductive development through alterations in the diurnal expression patterns of clock and flowering time genes. For this purpose, leaf samples of Scarlett and S42-IL107 plants grown under control and continuous mild drought conditions were harvested every 4 h over 24 h at the stamen primordium stage (≥W3.5).

**Fig. 5.**
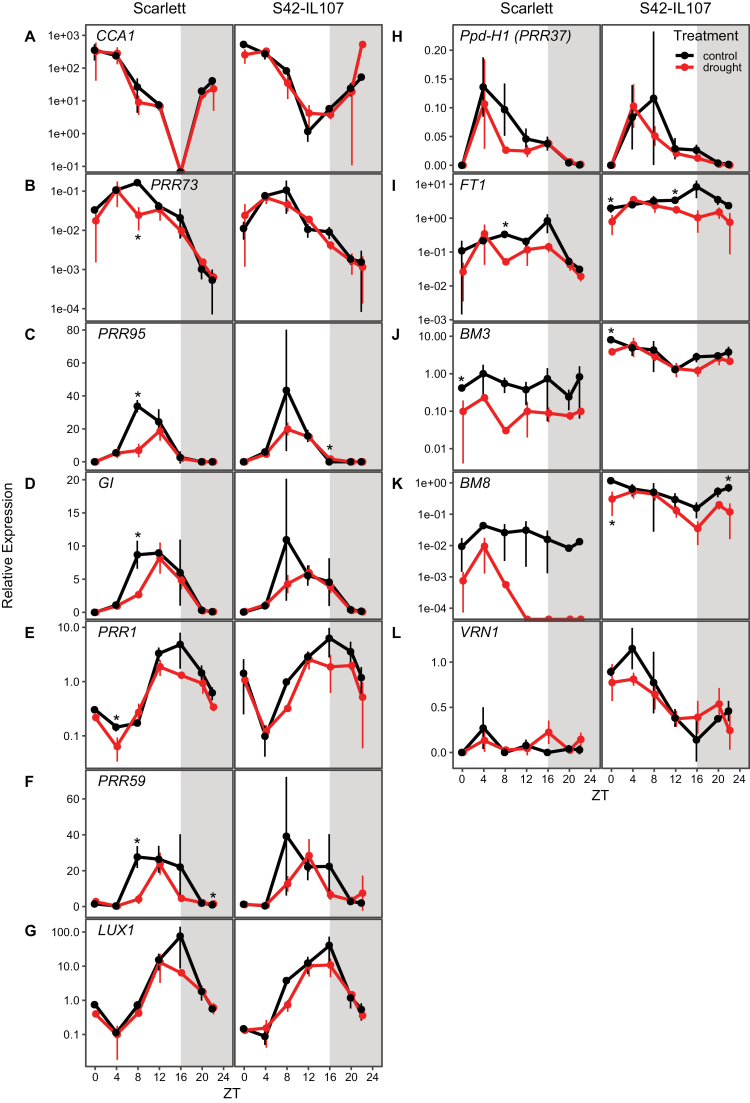
Continuous drought reduces the transcript levels of circadian clock and flowering time genes in barley. Diurnal gene expression of circadian clock genes was measured every 4 h for 24 h with an additional sampling at ZT22 under control (black) and drought (red) conditions under LDs (16 h light/8 h night) in the spring barley cultivars Scarlett and the derived introgression line S42-IL107. Grey areas indicate darkness. Error bars indicate ±SD of three biological replicates; an asterisk indicates a significant difference between control and drought at the respective time point (*t*-test, *P*<0.05).

We investigated the expression of known barley core clock genes (Campoli *et al.*, 2012b; Müller *et al.*, 2020), with expression peaks at different times of the day (Fig. 5). The expression levels of the morning-expressed *CCA1* and the evening-expressed *LUX1* were not consistently altered between drought and control conditions (Fig. 5A, G). Expression levels of *PRR59*, *PRR73*, *PRR95*, and *GIGANTEA* (*GI*) were down-regulated at ZT8 under drought compared with control conditions in Scarlett (Fig. 5B–D, F). Drought also affected the peak time of expression of some clock transcripts. The expression peaks of *PRR95* and *GI* were delayed by 4 h, while expression peaks of *PRR1* and *LUX1* were advanced by 4 h in both genotypes. There were no consistent differences in the expression levels and patterns of clock genes between Scarlett and S42-IL107 under both conditions. Similar to the clock genes, the floral regulator genes and putative downstream targets of *Ppd-H1* were down-regulated under drought. Expression of *Ppd-H1* itself was not strongly affected under drought in either genotype (Fig. 5H). However, the expression levels of floral regulator genes differed between the genotypes under control and drought conditions. Expression levels of *FT1*, and the barley MADS-box genes *VRN1*, *BM3*, and *BM8*, were overall higher in S42-IL107 than in Scarlett under both conditions (Fig. 5I–L). Drought reduced *FT1* transcript levels in both genotypes, in particular at the evening peak time of expression. However, expression of *FT1* under drought was at all time points higher in S42-IL107 than in Scarlett. *BM3* and *BM8* were down-regulated under drought specifically in Scarlett at the majority of time points (Fig. 5K). In S42-IL107, transcript levels of *BM3* and *BM8* were not strongly altered between control and drought conditions.

In summary, drought decreased the expression levels of clock genes and floral regulator genes, and affected the peak time of expression of evening-expressed clock genes. Expression patterns of clock genes were similar between Scarlett and S42-IL107 under control and drought conditions; genetic variation at *Ppd-H1* (*PRR37*) therefore did not affect the diel expression patterns of clock genes. However, expression of floral regulator genes was significantly different between Scarlett and S42-IL107 under control and drought conditions. In addition, expression levels of floral regulator genes were more strongly altered under drought in Scarlett than in S42-IL107, demonstrating that *Ppd-H1* interacted with drought to control reproductive development and expression levels of major flowering time genes in barley.

### *Ppd-H1* alters the effect of drought on flowering time gene expression during development

We further investigated how *Ppd-H1* and drought affected expression of floral regulator genes during development in all six genotypes. Transcript levels of floral regulator genes were investigated in leaf samples from plants analysed for developmental traits as shown in Fig. 2. The youngest fully developed leaf was harvested at ZT8 in all genotypes starting from the first day after transfer to LDs until flowering. At transfer to LDs, all genotypes had formed a reproductive inflorescence at the double ridge stage (W2), with the exception of BW281, which was already at the awn primordium stage (W3).

**Fig. 6.**
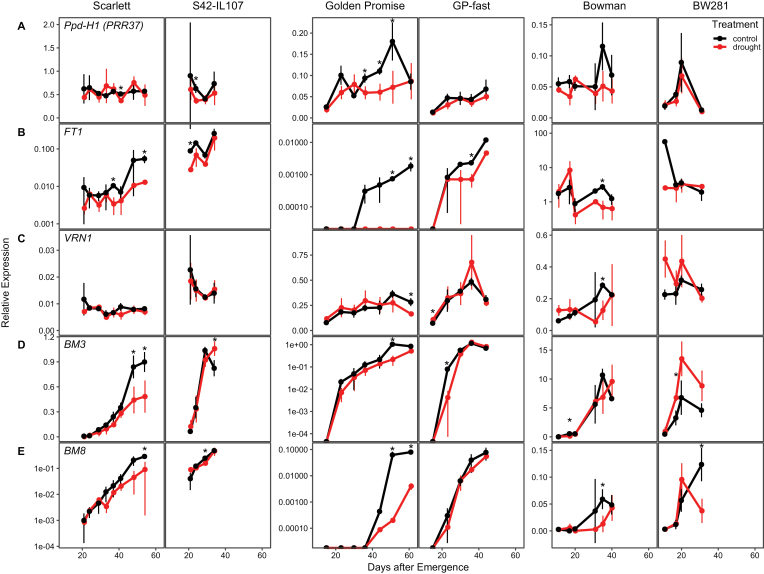
Continuous drought affects the expression of flowering time genes during development in barley. Transcript levels of flowering time genes was measured during development under control (black) and drought (red) conditions under LDs (16 h light/8 h night) in the spring barley cultivars Scarlett, Golden Promise, and Bowman, and the derived introgression lines S42-IL107, GP-fast, and BW281. Error bars indicate ±SD of three biological replicates; an asterisk indicates a significant difference between control and drought at the respective time point (*t*-test, *P*<0.05).

The expression levels of *Ppd-H1* were not strongly altered by the treatment or *Ppd-H1* variant, with the exception of Golden Promise where *Ppd-H1* transcript levels were significantly higher under control than drought conditions (W3.5–W5.5) (Fig. 6A). In contrast, *FT1* expression levels were down-regulated under drought in all genotypes (Fig. 6B). *FT1* transcript levels increased during development and this increase was slowed down under drought, in particular in the parental line Scarlett. In Golden Promise, no *FT1* transcript was detected under drought at any time point. In the introgression lines, *FT1* expression levels were only significantly different between conditions at single time points in S42-IL107 and GP-fast, and were not changed in BW281. These differences in *FT1* transcript levels under drought correlated with the observed delay in floral progression in the parental genotypes as compared with the introgression lines under drought versus control conditions (Fig. 6A). Transcript levels of the vernalization gene *VRN1* were higher in the introgression than parental lines, but not significantly different between control and drought conditions (Fig. 6C). Transcript levels of *BM3* and *BM8* increased during development in all genotypes, and this increase was delayed and reduced under drought in Scarlett, Golden Promise, and Bowman, but not significantly different in S42-IL107 and GP-fast under drought versus control treatments. In BW281, *BM3* expression levels increased faster and to higher levels under drought compared with control conditions which correlated with the acceleration in floral development under drought in this line (Fig. 6D, E).

**Fig. 7.**
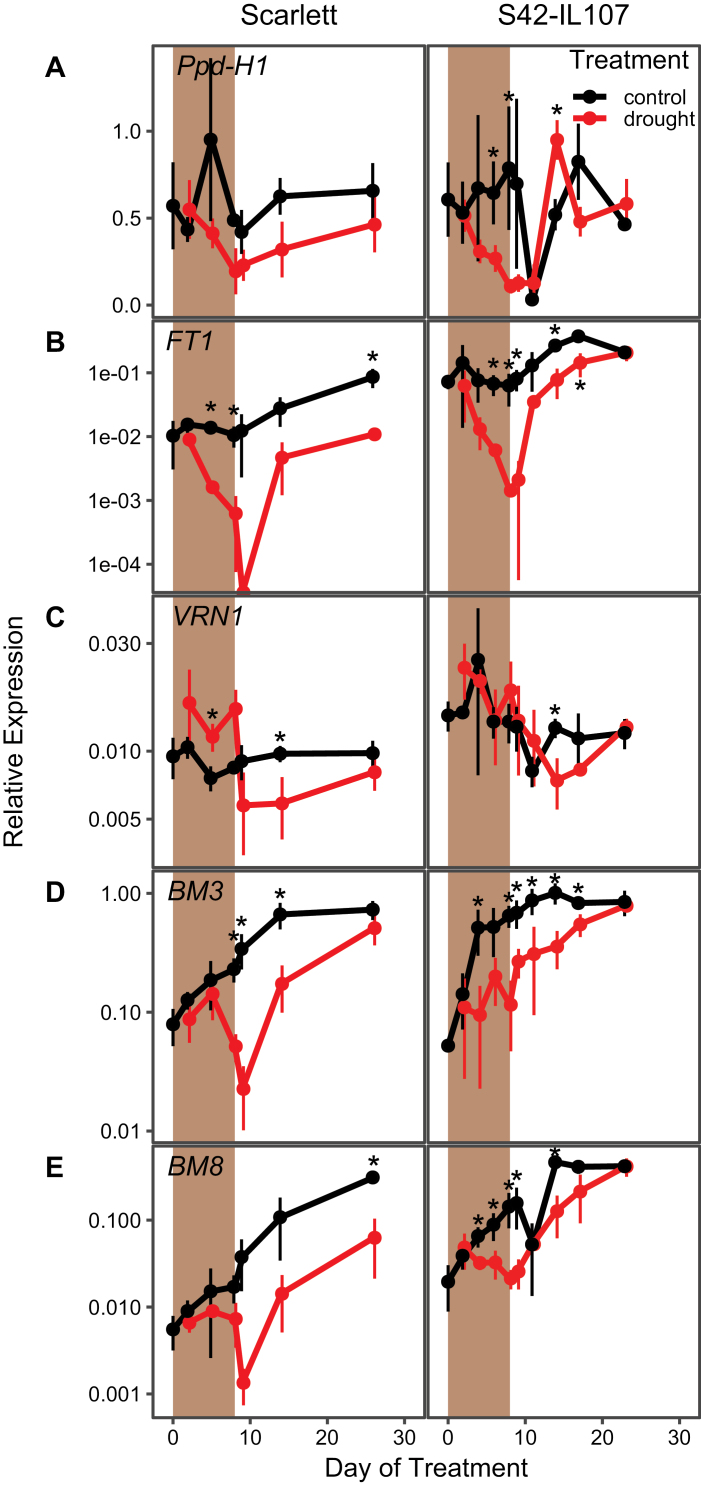
Severe drought interacts with *Ppd-H1* to control transcript levels of flowering time genes during stress and recovery. Transcript levels of flowering time genes were measured during development under control (black) and drought (red) conditions under LDs (16 h light/8 h night) in the spring barley cultivar Scarlett and the derived introgression line S42-IL107. Shaded areas indicate the period during which plants were not watered; error bars indicate ±SD of three biological replicates; an asterisk indicates a significant difference between control and drought at the respective time point (*t*-test, *P*<0.05).

We also tested the effects of the transient severe drought stress on the expression of floral regulator genes in Scarlett and S42-IL107 (Fig. 7). During the transient drought treatment, transcript levels of *Ppd-H1*, *FT1*, *BM3*, and *BM8* were strongly down-regulated compared with control conditions in both genotypes (Fig. 7A, B, D, E). In Scarlett, the down-regulation of these flowering inducers extended long into the recovery phase, even after leaf RWC had returned to control levels. In S42-IL107, transcript levels of floral inducers recovered rapidly after rewatering and eventually reached the same levels as observed under control conditions. Transcript levels of *VRN1* were down-regulated after the transient drought stress in both genotypes, but matched *VRN1* expression levels in control plants at flowering (Fig. 7C).

In summary, both mild continuous and severe transient drought reduced the transcript levels of flowering inducers. However, reductions in transcript levels were stronger in the parental than in the introgression lines with a wild-type *Ppd-H1* allele. *Ppd-H1* therefore modulated expression of floral inducers in response to drought in barley. In addition, transcript levels rapidly recovered after a transient drought stress to control levels in the introgression line but not the parental line, suggesting that *Ppd-H1* affected transcriptional homeostasis in response to drought.

## Discussion

*Ppd-H1* was identified as a photoperiod response gene that controls adaptation to different environments by modulating flowering time in response to LDs (Turner *et al.*, 2005; Cockram *et al.*, 2007; Jones *et al.*, 2008; Wiegmann *et al.*, 2019). Here, we demonstrate that *Ppd-H1* also integrates drought stress signals to modulate floral development in barley. Drought delayed floral development in the parental genotypes with a mutated *ppd-H1* allele, while reproductive development was not affected by drought in genotypes with a wild-type *Ppd-H1* allele (Figs 1, 2). This variation in developmental timing in response to drought was linked to variation in the number of initiated spikelet primordia on the main shoot. Spikelet initiation was reduced in the parental lines, but not in the introgression lines under drought (Fig. 3). Similarly, drought-triggered reductions in plant height, and tiller and spike number were more pronounced in the parental lines compared with the introgression lines. Under the severe transient stress, reproductive development slowed down in all genotypes; however, upon rewatering, the introgression line with a wild-type *Ppd-H1* allele accelerated development so that control and stressed plants flowered simultaneously. In contrast, parental lines flowered significantly later after a transient stress than plants under control conditions (Fig. 4). Taken together, the results demonstrated that *Ppd-H1* interacts with drought to control the development and morphology of the shoot and spike. *Ppd-H1* has already been associated with a number of shoot- and spike-related traits in barley and acts as a key gene to coordinate the development of different plant organs with reproductive timing (Digel et al., 2015, 2016; Alqudah et al., 2016, 2018; Ejaz and von Korff, 2017; Pham *et al.*, 2019; Shaaf *et al.*, 2019). Furthermore, our results suggested that *Ppd-H1* controlled the plasticity of reproductive development in response to drought. The parental lines with a mutation in *ppd*-*H1* displayed a high trait variance between treatments and thus developmental plasticity. In contrast, the introgression lines exhibited a higher trait stability under drought, in particular for developmental timing and spikelet initiation, while biomass reductions under drought were comparable between genotypes. The identification of genes/alleles maintaining trait stability in response to environmental perturbations is interesting for breeding genotypes with high yield stability under global climatic changes and higher frequencies of extreme weather events.

The most plastic trait under drought in all genotypes was grain number. Drought caused a minor reduction in the number of spikelet primordia and in the number of spikelets/florets, but a major reduction in the final grain number (Fig. 1). This suggested that drought reduced grain number primarily by affecting floret fertility and tiller number. It has already been described that water deficit impairs pollen development. Altered tapetal degeneration and associated changes in nutrient provision and signalling have been identified as the primary causes for cellular defects in pollen maturation under drought stress (Saini *et al.*, 1984; Lalonde *et al.*, 1997; Saini, 1997; Saini and Westgate, 1999; Aloni *et al.*, 2001; Pressman *et al.*, 2002; Barnabás *et al.*, 2008; De Storme and Geelen, 2014). Moreover, drought interferes with ovary survival or early grain development, potentially by restricting expansive growth, and thereby reduces the number of grains per spike (Guo *et al.*, 2016; Oury *et al.*, 2016a, b; Turc and Tardieu, 2018). In contrast to grain number, TKW was not very variable between drought and control conditions. We concluded that floral development is most susceptible to drought, while spikelet initiation as well as grain filling were less affected. These effects in controlled-environment chambers correspond to observations in field-grown wheat, where yield differences between environments were primarily controlled by variation in grain number while TKW was relatively stable across environments (Slafer et al., 2014, 2015). Our results therefore underline the importance of floral development and fertility for yield under drought, which supports recent studies that challenge the central importance of ‘terminal drought’ as the main cause for losses in cereal yield in drought-prone Mediterranean regions (Savin *et al.*, 2015).

The circadian clock controls genes of the photoperiod response pathway, and *Ppd-H1* itself is a barley homologue of an Arabidopsis clock gene (Faure *et al.*, 2012; Campoli *et al.*, 2013). Furthermore, the circadian clock controls stress adaptation and is itself regulated by stress cues (Liu *et al.*, 2013; Tamaru *et al.*, 2013; Habte *et al.*, 2014; Grundy *et al.*, 2015; Lee *et al.*, 2016; Ejaz and von Korff, 2017; Guadagno *et al.*, 2018). We consequently tested if drought interacted with variation at *Ppd-H1* to affect the expression of barley clock genes. Indeed, drought marginally affected the amplitude and phase of clock gene expression. Clock gene transcripts were down-regulated under drought; however, variation at *Ppd-H1* had no consistent effects on clock gene expression, under either control or drought conditions (Fig. 5). This supports earlier studies which demonstrated that the natural mutation in *ppd-H1* did not affect the expression of other barley clock homologues either under control conditions or under osmotic and/or high temperature stress (Campoli *et al.*, 2012b; Habte *et al.*, 2014; Ejaz and von Korff, 2017). However, we cannot exclude that drought might have interacted with *Ppd-H1* to affect clock proteins post-transcriptionally (Más *et al.*, 2003; Kiba *et al.*, 2007). Like the clock genes, the transcripts of the flowering time genes *FT1*, *BM3*, and *BM8* were reduced under drought during floral development (Figs 5, 6). Similarly, in rice, the *FT* homologues *Hd3a* and *RFT1* were down-regulated under drought stress and this correlated with a delay in floral transition under inductive SDs (Galbiati *et al.*, 2016). In contrast, in Arabidopsis, drought induces early flowering through the ABA-dependent stimulation of GI or of ABFs that trigger *SOC1* and *FT* transcriptional activation (Riboni *et al.*, 2016; Hwang *et al.*, 2019). On the other hand, it was also shown that ABSCISIC ACID-INSENSITIVE 4 (ABI4), a key component in the ABA signalling pathway, negatively regulated floral transition by directly promoting expression of the floral repressor *FLC*. Interestingly, the barley vernalization gene *VRN1* was not consistently altered in expression under drought, suggesting that the vernalization response pathway is not involved in transmitting drought signals in barley. However, all genotypes carry spring alleles at *VRN1*; future research therefore needs to test the response of the winter *vrn1* allele to drought and its effects on flowering.

Because negative and positive effects of drought and ABA on flowering time were observed, it was suggested that different levels of stress may elicit different developmental responses. A moderate level of drought and ABA levels may delay floral transition, allowing for flowering to occur after the stress, while a severe drought stress and high ABA levels promote flowering and drought escape to maximize reproductive success (Shu *et al.*, 2018). However, we found that both mild and severe stress resulted in a delay in flowering time. Differential responses to drought were rather genetically controlled where *Ppd-H1* controlled the drought-dependent down-regulation of *FT1*, *BM3*, and *BM8*, and correlated differences in reproductive development. Furthermore, after a transient drought stress, *FT1*, *BM3*, and *BM8* transcript levels recovered fast after rewatering and eventually matched those under control conditions in the introgression but not in the parental line (Fig. 7). Consequently, *Ppd-H1* also affected transcript homeostasis after a severe transient perturbation by stress. In contrast to reports from rice and Arabidopsis, drought did not strongly impact the timing of spikelet initiation but slowed down and impaired floral development and fertility. *FT1*, *BM3*, and *BM8* have already been linked to inflorescence and floral development in barley, wheat, and rice (Digel *et al.*, 2015; Wu *et al.*, 2017; Callens *et al.*, 2018; Shaw *et al.*, 2019). In rice, simultaneous knockdown of *OsMADS14* (*VRN1*, *FUL1*), *OsMADS15* (*BM3*, *FUL2*), and *OsMAD18* (*BM8*, *FUL3*) resulted in floral reversion and the formation of lateral vegetative tillers (Kobayashi *et al.*, 2012). Similarly, triple wheat *vrn1ful2ful3* mutants formed vegetative tillers instead of spikelets on lateral meristems and displayed a reduced stem elongation (Li *et al.*, 2019). Reduced transcript levels of *FT1*, *BM3*, and *BM8* might therefore have contributed to an impaired floral development and decreased stem elongation in the drought-stressed plants in our study. It has been shown in barley and rice, that *FT* homologues have positive effects on gibberellin (GA) biosynthesis or stem responsiveness to GA and thus stem elongation (Pearce *et al.*, 2013; Gómez-Ariza *et al.*, 2019). Reduced *FT1* transcript levels might therefore have contributed to a reduction in stem elongation under drought; Golden Promise with the strongest *FT1* down-regulation under drought was also characterized by the strongest reduction in plant height.

In summary, our results demonstrate that *Ppd-H1* integrates photoperiod and drought stress signals to control reproductive timing and the plasticity of shoot and spike morphology in response to drought in barley. These differential responses to drought are linked to a differential down-regulation of *FT1*, *BM3*, and *BM8* transcripts in the leaf. Future studies need to elucidate linked transcriptional changes in the inflorescences and further dissect the effects of drought on floral organ development. Furthermore, results obtained in this study under controlled conditions need to be verified under field conditions.

## Supplementary data

Supplementary data are available at *JXB* online.

**Table S1.** Oligonucleotides used in this study.

**Data S1.** Genotyping of introgression lines used in this study.

**Fig. S1.** Genetic map of *Ppd-H1* introgression in spring barley backgrounds, introgression size in centiMorgans (cM), and the polymorphic markers flanking the insertions.

**Fig. S2.** Flowering morphology in Golden Promise and Bowman background under continuous drought.

**Fig. S3.** MSA and pistil morphology in Golden Promise background.

**Fig. S4.** Continuous drought affects leaf size but not the phyllochron in barley.

**Fig. S5.** Severe drought delays flowering in barley.

eraa261_suppl_Supplementary_Dataset_S1

eraa261_suppl_Supplementary_Table_S1_Figures_S1-S5

## Abbreviations

ABA: abscisic acid
FC: field capacity
LD: long day
MSA: main shoot apex
RWC: relative water content
SAM: shoot apical meristem
SD: short day
SWC: soil water content

## References

[CIT0001] Abe M, Kobayashi Y, Yamamoto S, Daimon Y, Yamaguchi A, Ikeda Y, Ichinoki H, Notaguchi M, Goto K, Araki T 2005 FD, a bZIP protein mediating signals from the floral pathway integrator FT at the shoot apex. Science 309, 1052–1056. 16099979 10.1126/science.1115983

[CIT0002] Al-Ajlouni Z, Al-Abdallat A, Al-Ghzawi A, Ayad J, Abu Elenein J, Al-Quraan N, Baenziger P 2016 Impact of pre-anthesis water deficit on yield and yield components in barley (*Hordeum vulgare* L.) plants grown under controlled conditions. Agronomy 6, 33.

[CIT0003] Aloni B, Peet M, Pharr M, Karni L 2001 The effect of high temperature and high atmospheric CO_2_ on carbohydrate changes in bell pepper (*Capsicum annuum*) pollen in relation to its germination. Physiologia Plantarum 112, 505–512. 11473710 10.1034/j.1399-3054.2001.1120407.x

[CIT0004] Alqudah AM, Koppolu R, Wolde GM, Graner A, Schnurbusch T 2016 The genetic architecture of barley plant stature. Frontiers in Genetics 7, 117. 27446200 10.3389/fgene.2016.00117 PMC4919324

[CIT0005] Alqudah AM, Schnurbusch T 2017 Heading date is not flowering time in spring barley. Frontiers in Plant Science 8, 896. 28611811 10.3389/fpls.2017.00896 PMC5447769

[CIT0006] Alqudah AM, Sharma R, Pasam RK, Graner A, Kilian B, Schnurbusch T 2014 Genetic dissection of photoperiod response based on GWAS of pre-anthesis phase duration in spring barley. PLoS One 9, e113120. 25420105 10.1371/journal.pone.0113120 PMC4242610

[CIT0007] Alqudah AM, Youssef HM, Graner A, Schnurbusch T 2018 Natural variation and genetic make-up of leaf blade area in spring barley. Theoretical and Applied Genetics 131, 873–886. 29350248 10.1007/s00122-018-3053-2 PMC5852197

[CIT0008] Alvarez MA, Tranquilli G, Lewis S, Kippes N, Dubcovsky J 2016 Genetic and physical mapping of the earliness per se locus *Eps-A (m) 1* in *Triticum monococcum* identifies *EARLY FLOWERING 3* (*ELF3*) as a candidate gene. Functional & Integrative Genomics 16, 365–382. 27085709 10.1007/s10142-016-0490-3 PMC4947483

[CIT0009] Barnabás B, Jäger K, Fehér A 2008 The effect of drought and heat stress on reproductive processes in cereals. Plant, Cell & Environment 31, 11–38. 10.1111/j.1365-3040.2007.01727.x 17971069

[CIT0010] Baum M, Von Korff M, Guo P, et al 2007 Molecular approaches and breeding strategies for drought tolerance in barley. In: Varshney R, Tuberosa R, eds. Genomic assisted crop improvement: Vol. 2: Genomics applications in crops. Dordrecht: Springer, 51–79.

[CIT0011] Bayer MM, Rapazote-Flores P, Ganal M, et al 2017 Development and evaluation of a barley 50k iSelect SNP array. Frontiers in Plant Science 8, 1792. 29089957 10.3389/fpls.2017.01792 PMC5651081

[CIT0012] Callens C, Tucker MR, Zhang D, Wilson ZA 2018 Dissecting the role of MADS-box genes in monocot floral development and diversity. Journal of Experimental Botany 69, 2435–2459. 29718461 10.1093/jxb/ery086

[CIT0013] Campoli C, Drosse B, Searle I, Coupland G, von Korff M 2012 *a* Functional characterisation of *HvCO1*, the barley (*Hordeum vulgare*) flowering time ortholog of CONSTANS. The Plant Journal 69, 868–880. 22040323 10.1111/j.1365-313X.2011.04839.x

[CIT0014] Campoli C, Pankin A, Drosse B, Casao CM, Davis SJ, von Korff M 2013 *HvLUX1* is a candidate gene underlying the *early maturity* 10 locus in barley: phylogeny, diversity, and interactions with the circadian clock and photoperiodic pathways. New Phytologist 199, 1045–1059. 10.1111/nph.12346 PMC3902989 23731278

[CIT0015] Campoli C, Shtaya M, Davis SJ, von Korff M 2012 *b* Expression conservation within the circadian clock of a monocot: natural variation at barley *Ppd-H1* affects circadian expression of flowering time genes, but not clock orthologs. BMC Plant Biology 12, 97. 22720803 10.1186/1471-2229-12-97 PMC3478166

[CIT0016] Cantalapiedra CP, Boudiar R, Casas AM, Igartua E, Contreras-Moreira B 2015 BARLEYMAP: physical and genetic mapping of nucleotide sequences and annotation of surrounding loci in barley. Molecular Breeding 35, 13.

[CIT0017] Cockram J, Jones H, Leigh FJ, O’Sullivan D, Powell W, Laurie DA, Greenland AJ 2007 Control of flowering time in temperate cereals: genes, domestication, and sustainable productivity. Journal of Experimental Botany 58, 1231–1244. 17420173 10.1093/jxb/erm042

[CIT0018] Coleman E 1947 A laboratory procedure for determining the field capacity of soils. Soil Science 63, 277–284.

[CIT0019] Corbesier L, Vincent C, Jang S, et al 2007 FT protein movement contributes to long-distance signaling in floral induction of *Arabidopsis*. Science 316, 1030–1033. 17446353 10.1126/science.1141752

[CIT0020] De Storme N, Geelen D 2014 The impact of environmental stress on male reproductive development in plants: biological processes and molecular mechanisms. Plant, Cell & Environment 37, 1–18. 10.1111/pce.12142 PMC4280902 23731015

[CIT0021] Digel B, Pankin A, von Korff M 2015 Global transcriptome profiling of developing leaf and shoot apices reveals distinct genetic and environmental control of floral transition and inflorescence development in barley. The Plant Cell 27, 2318–2334. 26307377 10.1105/tpc.15.00203 PMC4815099

[CIT0022] Digel B, Tavakol E, Verderio G, Tondelli A, Xu X, Cattivelli L, Rossini L, von Korff M 2016 Photoperiod-H1 (Ppd-H1) controls leaf size. Plant Physiology 172, 405–415. 27457126 10.1104/pp.16.00977 PMC5074620

[CIT0023] Druka A, Franckowiak J, Lundqvist U, et al 2011 Genetic dissection of barley morphology and development. Plant Physiology 155, 617–627. 21088227 10.1104/pp.110.166249 PMC3032454

[CIT0024] Ejaz M, von Korff M 2017 The genetic control of reproductive development under high ambient temperature. Plant Physiology 173, 294–306. 28049855 10.1104/pp.16.01275 PMC5210726

[CIT0025] Faure S, Turner AS, Gruszka D, Christodoulou V, Davis SJ, von Korff M, Laurie DA 2012 Mutation at the circadian clock gene *EARLY MATURITY 8* adapts domesticated barley (*Hordeum vulgare*) to short growing seasons. Proceedings of the National Academy of Sciences, USA 109, 8328–8333. 10.1073/pnas.1120496109 PMC3361427 22566625

[CIT0026] Ford B, Deng W, Clausen J, Oliver S, Boden S, Hemming M, Trevaskis B 2016 Barley (*Hordeum vulgare*) circadian clock genes can respond rapidly to temperature in an *EARLY FLOWERING 3*-dependent manner. Journal of Experimental Botany 67, 5517–5528. 27580625 10.1093/jxb/erw317 PMC5049398

[CIT0027] Galbiati F, Chiozzotto R, Locatelli F, Spada A, Genga A, Fornara F 2016 *Hd3a, RFT1* and *Ehd1* integrate photoperiodic and drought stress signals to delay the floral transition in rice. Plant, Cell & Environment 39, 1982–1993. 10.1111/pce.12760 27111837

[CIT0028] Gol L, Tomé F, von Korff M 2017 Floral transitions in wheat and barley: interactions between photoperiod, abiotic stresses, and nutrient status. Journal of Experimental Botany 68, 1399–1410. 28431134 10.1093/jxb/erx055

[CIT0029] Gómez-Ariza J, Brambilla V, Vicentini G, et al 2019 A transcription factor coordinating internode elongation and photoperiodic signals in rice. Nature Plants 5, 358–362. 30936438 10.1038/s41477-019-0401-4

[CIT0030] Grundy J, Stoker C, Carré IA 2015 Circadian regulation of abiotic stress tolerance in plants. Frontiers in Plant Science 6, 648. 26379680 10.3389/fpls.2015.00648 PMC4550785

[CIT0031] Guadagno CR, Ewers BE, Weinig C 2018 Circadian rhythms and redox state in plants: till stress do us part. Frontiers in Plant Science 9, 247. 29556244 10.3389/fpls.2018.00247 PMC5844964

[CIT0032] Guo Z, Slafer GA, Schnurbusch T 2016 Genotypic variation in spike fertility traits and ovary size as determinants of floret and grain survival rate in wheat. Journal of Experimental Botany 67, 4221–4230. 27279276 10.1093/jxb/erw200 PMC5301927

[CIT0033] Habte E, Müller LM, Shtaya M, Davis SJ, von Korff M 2014 Osmotic stress at the barley root affects expression of circadian clock genes in the shoot. Plant, Cell & Environment 37, 1321–1327. 10.1111/pce.12242 24895755

[CIT0034] Hwang K, Susila H, Nasim Z, Jung JY, Ahn JH 2019 *Arabidopsis* ABF3 and ABF4 transcription factors act with the NF-YC complex to regulate *SOC1* expression and mediate drought-accelerated flowering. Molecular Plant 12, 489–505. 30639313 10.1016/j.molp.2019.01.002

[CIT0035] Jaeger KE, Pullen N, Lamzin S, Morris RJ, Wigge PA 2013 Interlocking feedback loops govern the dynamic behavior of the floral transition in *Arabidopsis*. The Plant Cell 25, 820–833. 23543784 10.1105/tpc.113.109355 PMC3634691

[CIT0036] Jaeger KE, Wigge PA 2007 FT protein acts as a long-range signal in *Arabidopsis*. Current Biology 17, 1050–1054. 17540569 10.1016/j.cub.2007.05.008

[CIT0037] Johansson M, Staiger D 2015 Time to flower: interplay between photoperiod and the circadian clock. Journal of Experimental Botany 66, 719–730. 25371508 10.1093/jxb/eru441

[CIT0038] Jones H, Leigh FJ, Mackay I, Bower MA, Smith LM, Charles MP, Jones G, Jones MK, Brown TA, Powell W 2008 Population-based resequencing reveals that the flowering time adaptation of cultivated barley originated east of the Fertile Crescent. Molecular Biology and Evolution 25, 2211–2219. 18669581 10.1093/molbev/msn167

[CIT0039] Kahiluoto H, Kaseva J, Balek J, et al 2019 Decline in climate resilience of European wheat. Proceedings of the National Academy of Sciences, USA 116, 123–128. 10.1073/pnas.1804387115 PMC6320549 30584094

[CIT0040] Kazan K, Lyons R 2016 The link between flowering time and stress tolerance. Journal of Experimental Botany 67, 47–60. 26428061 10.1093/jxb/erv441

[CIT0041] Kiba T, Henriques R, Sakakibara H, Chua NH 2007 Targeted degradation of PSEUDO-RESPONSE REGULATOR5 by an SCFZTL complex regulates clock function and photomorphogenesis in *Arabidopsis thaliana*. The Plant Cell 19, 2516–2530. 17693530 10.1105/tpc.107.053033 PMC2002626

[CIT0042] Kobayashi K, Yasuno N, Sato Y, Yoda M, Yamazaki R, Kimizu M, Yoshida H, Nagamura Y, Kyozuka J 2012 Inflorescence meristem identity in rice is specified by overlapping functions of three *AP1/FUL*-like MADS box genes and *PAP2*, a *SEPALLATA* MADS box gene. The Plant Cell 24, 1848–1859. 22570445 10.1105/tpc.112.097105 PMC3442573

[CIT0043] Lalonde S, Beebe DU, Saini HS 1997 Early signs of disruption of wheat anther development associated with the induction of male sterility by meiotic-stage water deficit. Sexual Plant Reproduction 10, 40–48.

[CIT0044] Lee HG, Mas P, Seo PJ 2016 MYB96 shapes the circadian gating of ABA signaling in *Arabidopsis*. Scientific Reports 6, 17754. 26725725 10.1038/srep17754 PMC4698719

[CIT0045] Li C, Lin H, Chen A, Lau M, Jernstedt J, Dubcovsky J 2019 Wheat VRN1, FUL2 and FUL3 play critical and redundant roles in spikelet development and spike determinacy. Development 146, dev175398. 31337701 10.1242/dev.175398 PMC6679363

[CIT0046] Liu T, Carlsson J, Takeuchi T, Newton L, Farré EM 2013 Direct regulation of abiotic responses by the Arabidopsis circadian clock component PRR7. The Plant Journal 76, 101–114. 23808423 10.1111/tpj.12276

[CIT0047] Makino S, Matsushika A, Kojima M, Oda Y, Mizuno T 2001 Light response of the circadian waves of the APRR1/TOC1 quintet: when does the quintet start singing rhythmically in Arabidopsis? Plant & Cell Physiology 42, 334–339. 11266585 10.1093/pcp/pce036

[CIT0048] Más P, Kim WY, Somers DE, Kay SA 2003 Targeted degradation of TOC1 by ZTL modulates circadian function in *Arabidopsis thaliana*. Nature 426, 567–570. 14654842 10.1038/nature02163

[CIT0049] Mascher M, Gundlach H, Himmelbach A, et al 2017 A chromosome conformation capture ordered sequence of the barley genome. Nature 544, 427–433. 28447635 10.1038/nature22043

[CIT0050] Mathieu J, Warthmann N, Küttner F, Schmid M 2007 Export of FT protein from phloem companion cells is sufficient for floral induction in *Arabidopsis*. Current Biology 17, 1055–1060. 17540570 10.1016/j.cub.2007.05.009

[CIT0051] Mizuno N, Nitta M, Sato K, Nasuda S 2012 A wheat homologue of *PHYTOCLOCK 1* is a candidate gene conferring the early heading phenotype to einkorn wheat. Genes & Genetic Systems 87, 357–367. 23558642 10.1266/ggs.87.357

[CIT0052] Müller L, Mombaerts L, Pankin A, Davis SJ, Webb AA, Goncalves J, von Korff M 2020 Differential effects of day–night cues and the circadian clock on the barley transcriptome. Plant Physiology (in press). 10.1104/pp.19.01411 PMC7271788 32229608

[CIT0053] Müller LM, von Korff M, Davis SJ 2014 Connections between circadian clocks and carbon metabolism reveal species-specific effects on growth control. Journal of Experimental Botany 65, 2915–2923. 24706717 10.1093/jxb/eru117

[CIT0054] Murakami M, Ashikari M, Miura K, Yamashino T, Mizuno T 2003 The evolutionarily conserved *O*sPRR quintet: rice pseudo-response regulators implicated in circadian rhythm. Plant & Cell Physiology 44, 1229–1236. 14634161 10.1093/pcp/pcg135

[CIT0055] Oury V, Caldeira CF, Prodhomme D, Pichon JP, Gibon Y, Tardieu F, Turc O 2016 Is change in ovary carbon status a cause or a consequence of maize ovary abortion in water deficit during flowering? Plant Physiology 171, 997–1008. 27208256 10.1104/pp.15.01130 PMC4902574

[CIT0056] Oury V, Tardieu F, Turc O 2016 Ovary apical abortion under water deficit is caused by changes in sequential development of ovaries and in silk growth rate in maize. Plant Physiology 171, 986–996. 26598464 10.1104/pp.15.00268 PMC4902573

[CIT0057] Pearce S, Vanzetti LS, Dubcovsky J 2013 Exogenous gibberellins induce wheat spike development under short days only in the presence of *VERNALIZATION1*. Plant Physiology 163, 1433–1445. 24085801 10.1104/pp.113.225854 PMC3813662

[CIT0058] Pham AT, Maurer A, Pillen K, Brien C, Dowling K, Berger B, Eglinton JK, March TJ 2019 Genome-wide association of barley plant growth under drought stress using a nested association mapping population. BMC Plant Biology 19, 134. 30971212 10.1186/s12870-019-1723-0 PMC6458831

[CIT0059] Pressman E, Peet MM, Pharr DM 2002 The effect of heat stress on tomato pollen characteristics is associated with changes in carbohydrate concentration in the developing anthers. Annals of Botany 90, 631–636. 12466104 10.1093/aob/mcf240 PMC4240456

[CIT0060] R Core Team. 2020 R: a language and environment for statistical computing. Vienna, Austria: R Foundation for Statistical Computing.

[CIT0061] Riboni M, Galbiati M, Tonelli C, Conti L 2013 *GIGANTEA* enables drought escape response via abscisic acid-dependent activation of the florigens and *SUPPRESSOR OF OVEREXPRESSION OF CONSTANS*. Plant Physiology 162, 1706–1719. 23719890 10.1104/pp.113.217729 PMC3707542

[CIT0062] Riboni M, Robustelli Test A, Galbiati M, Tonelli C, Conti L 2016 ABA-dependent control of GIGANTEA signalling enables drought escape via up-regulation of *FLOWERING LOCUS T* in *Arabidopsis thaliana*. Journal of Experimental Botany 67, 6309–6322. 27733440 10.1093/jxb/erw384 PMC5181575

[CIT0063] Rollins JA, Drosse B, Mulki MA, Grando S, Baum M, Singh M, Ceccarelli S, von Korff M 2013 *a* Variation at the vernalisation genes *Vrn-H1* and *Vrn-H2* determines growth and yield stability in barley (*Hordeum vulgare*) grown under dryland conditions in Syria. Theoretical and Applied Genetics 126, 2803–2824. 23918065 10.1007/s00122-013-2173-y

[CIT0064] Rollins JA, Habte E, Templer SE, Colby T, Schmidt J, von Korff M 2013 *b* Leaf proteome alterations in the context of physiological and morphological responses to drought and heat stress in barley (*Hordeum vulgare* L.). Journal of Experimental Botany 64, 3201–3212. 23918963 10.1093/jxb/ert158 PMC3733145

[CIT0065] Saini HS 1997 Effects of water stress on male gametophyte development in plants. Sexual Plant Reproduction 10, 67–73.

[CIT0066] Saini H, Sedgley M, Aspinall D 1984 Development anatomy in wheat of male sterility induced by heat stress, water deficit or abscisic acid. Functional Plant Biology 11, 243.

[CIT0067] Saini HS, Westgate ME 1999 Reproductive development in grain crops during drought. Advances in Agronomy 68, 59–96.

[CIT0068] Sanchez A, Shin J, Davis SJ 2011 Abiotic stress and the plant circadian clock. Plant Signaling & Behavior 6, 223–231. 21325898 10.4161/psb.6.2.14893 PMC3121982

[CIT0069] Savin R, Slafer GA, Cossani CM, Abeledo LG 2015 Cereal yield in Mediterranean-type environments: challenging the paradigms on terminal drought, the adaptability of barley vs wheat and the role of nitrogen fertilization. In: Sadras VO, Calderini DF, eds. Crop physiology. Applications for genetic improvement and agronomy, 2nd edn Academic Press, 141–158.

[CIT0070] Schmalenbach I, March TJ, Bringezu T, Waugh R, Pillen K 2011 High-resolution genotyping of wild barley introgression lines and fine-mapping of the threshability locus *thresh-1* using the illumina goldengate assay. G3 (Bethesda, Md.) 1, 187–196. 10.1534/g3.111.000182 PMC3276139 22384330

[CIT0071] Schmitz J, Franzen R, Ngyuen TH, Garcia-Maroto F, Pozzi C, Salamini F, Rohde W 2000 Cloning, mapping and expression analysis of barley MADS-box genes. Plant Molecular Biology 42, 899–913. 10890536 10.1023/a:1006425619953

[CIT0072] Shaaf S, Bretani G, Biswas A, Fontana IM, Rossini L 2019 Genetics of barley tiller and leaf development. Journal of Integrative Plant Biology 61, 226–256. 30548413 10.1111/jipb.12757

[CIT0073] Shaw LM, Lyu B, Turner R, Li C, Chen F, Han X, Fu D, Dubcovsky J 2019 *FLOWERING LOCUS T2* regulates spike development and fertility in temperate cereals. Journal of Experimental Botany 70, 193–204. 30295847 10.1093/jxb/ery350 PMC6305198

[CIT0074] Shu K, Chen Q, Wu Y, Liu R, Zhang H, Wang S, Tang S, Yang W, Xie Q 2016 ABSCISIC ACID-INSENSITIVE 4 negatively regulates flowering through directly promoting Arabidopsis *FLOWERING LOCUS C* transcription. Journal of Experimental Botany 67, 195–205. 26507894 10.1093/jxb/erv459 PMC4682436

[CIT0075] Shu K, Luo X, Meng Y, Yang W 2018 Toward a molecular understanding of abscisic acid actions in floral transition. Plant & Cell Physiology 59, 215–221. 29361058 10.1093/pcp/pcy007

[CIT0076] Slafer GA, Kantolic AG, Appendino ML, Tranquilli G, Miralles DJ, Savin R 2015 Genetic and environmental effects on crop development determining adaptation and yield. In: Sadras VO, Calderini DF, eds. Crop physiology. Applications for genetic improvement and agronomy, 2nd edn Academic Press, 285–319.

[CIT0077] Slafer GA, Savin R, Sadras VO 2014 Coarse and fine regulation of wheat yield components in response to genotype and environment. Field Crops Research 157, 71–83.

[CIT0078] Smart RE, Bingham GE 1974 Rapid estimates of relative water content. Plant Physiology 53, 258–260. 16658686 10.1104/pp.53.2.258 PMC541374

[CIT0079] Tamaki S, Matsuo S, Wong HL, Yokoi S, Shimamoto K 2007 Hd3a protein is a mobile flowering signal in rice. Science 316, 1033–1036. 17446351 10.1126/science.1141753

[CIT0080] Tamaru T, Hattori M, Ninomiya Y, et al 2013 ROS stress resets circadian clocks to coordinate pro-survival signals. PLoS One 8, e82006. 24312621 10.1371/journal.pone.0082006 PMC3846904

[CIT0081] Templer SE, Ammon A, Pscheidt D, et al 2017 Metabolite profiling of barley flag leaves under drought and combined heat and drought stress reveals metabolic QTLs for metabolites associated with antioxidant defense. Journal of Experimental Botany 68, 1697–1713. 28338908 10.1093/jxb/erx038 PMC5441916

[CIT0082] Trevaskis B, Tadege M, Hemming MN, Peacock WJ, Dennis ES, Sheldon C 2007 *Short vegetative phase-like* MADS-box genes inhibit floral meristem identity in barley. Plant Physiology 143, 225–235. 17114273 10.1104/pp.106.090860 PMC1761976

[CIT0083] Turc O, Tardieu F 2018 Drought affects abortion of reproductive organs by exacerbating developmentally driven processes via expansive growth and hydraulics. Journal of Experimental Botany 69, 3245–3254. 29546424 10.1093/jxb/ery078

[CIT0084] Turner A, Beales J, Faure S, Dunford RP, Laurie DA 2005 The pseudo-response regulator *Ppd-H1* provides adaptation to photoperiod in barley. Science 310, 1031–1034. 16284181 10.1126/science.1117619

[CIT0085] von Korff M, Grando S, Del Greco A, This D, Baum M, Ceccarelli S 2008 Quantitative trait loci associated with adaptation to Mediterranean dryland conditions in barley. Theoretical and Applied Genetics 117, 653–669. 18618094 10.1007/s00122-008-0787-2

[CIT0086] Waddington SR, Cartwright PM, Wall PC 1983 A quantitative scale of spike initial and pistil development in barley and wheat. Annals of Botany 51, 119–130.

[CIT0087] Wang Y, Li L, Ye T, Lu Y, Chen X, Wu Y 2013 The inhibitory effect of ABA on floral transition is mediated by ABI5 in *Arabidopsis*. Journal of Experimental Botany 64, 675–684. 23307919 10.1093/jxb/ers361 PMC3542054

[CIT0088] Wiegmann M, Maurer A, Pham A, et al 2019 Barley yield formation under abiotic stress depends on the interplay between flowering time genes and environmental cues. Scientific Reports 9, 6397. 31024028 10.1038/s41598-019-42673-1 PMC6484077

[CIT0089] Wigge PA, Kim MC, Jaeger KE, Busch W, Schmid M, Lohmann JU, Weigel D 2005 Integration of spatial and temporal information during floral induction in *Arabidopsis*. Science 309, 1056–1059. 16099980 10.1126/science.1114358

[CIT0090] Wu F, Shi X, Lin X, Liu Y, Chong K, Theißen G, Meng Z 2017 The ABCs of flower development: mutational analysis of *AP1/FUL*-like genes in rice provides evidence for a homeotic (A)-function in grasses. The Plant Journal 89, 310–324. 27689766 10.1111/tpj.13386

[CIT0091] Xie W, Xiong W, Pan J, Ali T, Cui Q, Guan D, Meng J, Mueller ND, Lin E, Davis SJ 2018 Decreases in global beer supply due to extreme drought and heat. Nature Plants 4, 964–973. 30323183 10.1038/s41477-018-0263-1

[CIT0092] Zadoks JC, Chang TT, Konzak CF 1974 A decimal code for the growth stages of cereals. Weed Research 14, 415–421.

[CIT0093] Zakhrabekova S, Gough SP, Braumann I, et al 2012 Induced mutations in circadian clock regulator *Mat-a* facilitated short-season adaptation and range extension in cultivated barley. Proceedings of the National Academy of Sciences, USA 109, 4326–4331. 10.1073/pnas.1113009109 PMC3306670 22371569

[CIT0094] Zhang C, Liu J, Zhao T, et al 2016 A drought-inducible transcription factor delays reproductive timing in rice. Plant Physiology 171, 334–343. 26945049 10.1104/pp.16.01691 PMC4854678

