# Supplementary material for: Ppd-H1 integrates drought stress signals to control spike development and flowering time in barley
Source: J Exp Bot. 2020 May 27;72(1):122–36. doi: 10.1093/jxb/eraa261 (PMC7816852; doi:10.1093/jxb/eraa261)
Supplement: eraa261_suppl_Supplementary_Table_S1_Figures_S1-S5 [file eraa261_suppl_supplementary_table_s1_figures_s1-s5.pdf]

Supplemental Table 1: Oligonucleotides used in this study

Gene ID	Gene name	Forward primer sequence	Reverse primer sequence	Source
AJ249143	*HvBM3*	GCC GTC ACC AGC ACA AGC AA	CCC CAT TCA CCC TGT AGC AAA GA	Digel et al., 2015
AJ249146	*HvBM8*	CCA CAG CAG CCG ACA CCT A	TGC CTT TGG GGG AGA AGA CG	Digel et al., 2015
AK362208	*HvGAPDH*	GTG AGG CTG GTG CTG ATT ACG	AGT GGT GCA GCT AGC ATT TGA GAC	Ejaz and von Korff, 2016
AK376549	*HvPRR73*	GCG CCG TAG AGA ATC AGA AC	CAT GTC GGG TAC AGT CAT CG	Campoli et al., 2012b
AY145451	*HvACTIN*	CGT GTT GGA TTC TGG TGA TG	AGC CAC ATA TGC GAG CTT CT	Campoli et al., 2012a
AY740524	*HvGI*	TCA GTT AGA GCT CCT GGA AGT	GGT AGT TTG GGC TTT GGA TG	Campoli et al., 2012b
AY750995	*HvVRN1*	CTG AAG GCG AAG GTT GAG AC	TTC TCC TCC TGC AGT GAC CT	Campoli et al., 2012a
AY970701	*Ppd-H1*	GAT GGA TTC AAA GGC AAG GA	GAA CAA TTG GCT CCT CCA AA	Campoli et al., 2012a
DQ100327	*HvFT1*	GGT AGA CCC AGA TGC TCC AA	TCG TAG CAC ATC ACC TCC TG	Campoli et al., 2012a
AY150676	*HvABI5*	CGC GCT GAA GTA TTG AAA CA	CAC CAG AAC GTT GCA GCT TA	Kobayashi et al., 2008
AJ508228.2	*HvADP*	GCT CTC CAA CAA CAT TGC CAA C	GAG ACA TCC AGC ATC ATT CAT TCC	Ferdous et al., 2015
JN603243	*HvPRR1*	GAG CAT AGC ATG GCA CTT CA	TGT CTT TCC TCG GAA ATT GG	Campoli et al.2012b
AK361360	*HvPRR59*	GAA ATT CCG CAT GAA AAG GA	TTC CGC ATC TTC TGT TGT TG	Campoli et al.2012b
JN603242	*HvCCA1*	CCT GGA ATT GGA GAT GGA GA	TGA GCA TGG CTT CTG ATT TG	Campoli et al.2012b
AK252005	*HvPRR95*	CAG AAC TCC AGT GTC GCA AA	TGC TGT TGC CAG AGT TGT TC	Campoli et al.2012b
Hv.20312	*HvLUX1*	AAT TCA GTC CAC GGA TGC TC	CTT CAC TTC AGC TCC CCT TG	Campoli et al.2012b

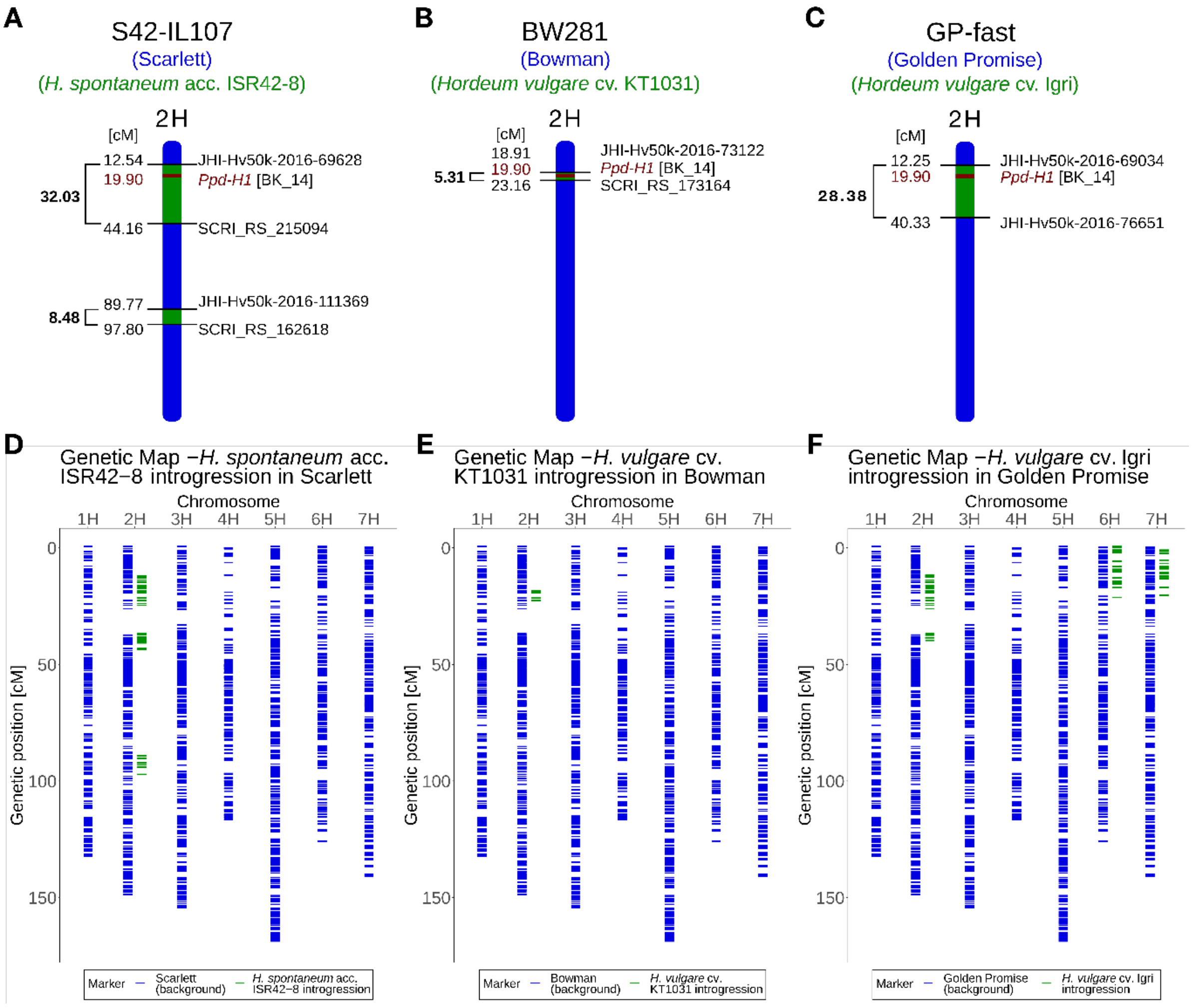

Supplemental Figure 1 Genetic map of *Ppd-H1* introgression in spring barley backgrounds, introgression size in centimorgan (cM) and the polymorphic markers flanking the insertions. Donor parents of wild-type *Ppd-H1* allele (green) introgressed into their respective background cultivars (blue). Introgression lines were genotyped with the Barley 50k iSelect SNP Array (Bayer *et al.*, 2017). Location of introgressed *Ppd-H1* allele and flanking regions on chromosome 2H (A-C), and overview of all chromosomes of the three introgression lines (D-F). Mapping positions of markers were obtained from the POPSEQ_2017 map (Cantalapiedra *et al.*, 2015; Mascher *et al.*, 2017), and the sizes of introgressed regions were calculated from the midway point between flanking SNPs. There are additional donor introgressions in GP-fast on chromosome 6H and 7H, 22.34 cM and 19.90 cM, respectively.

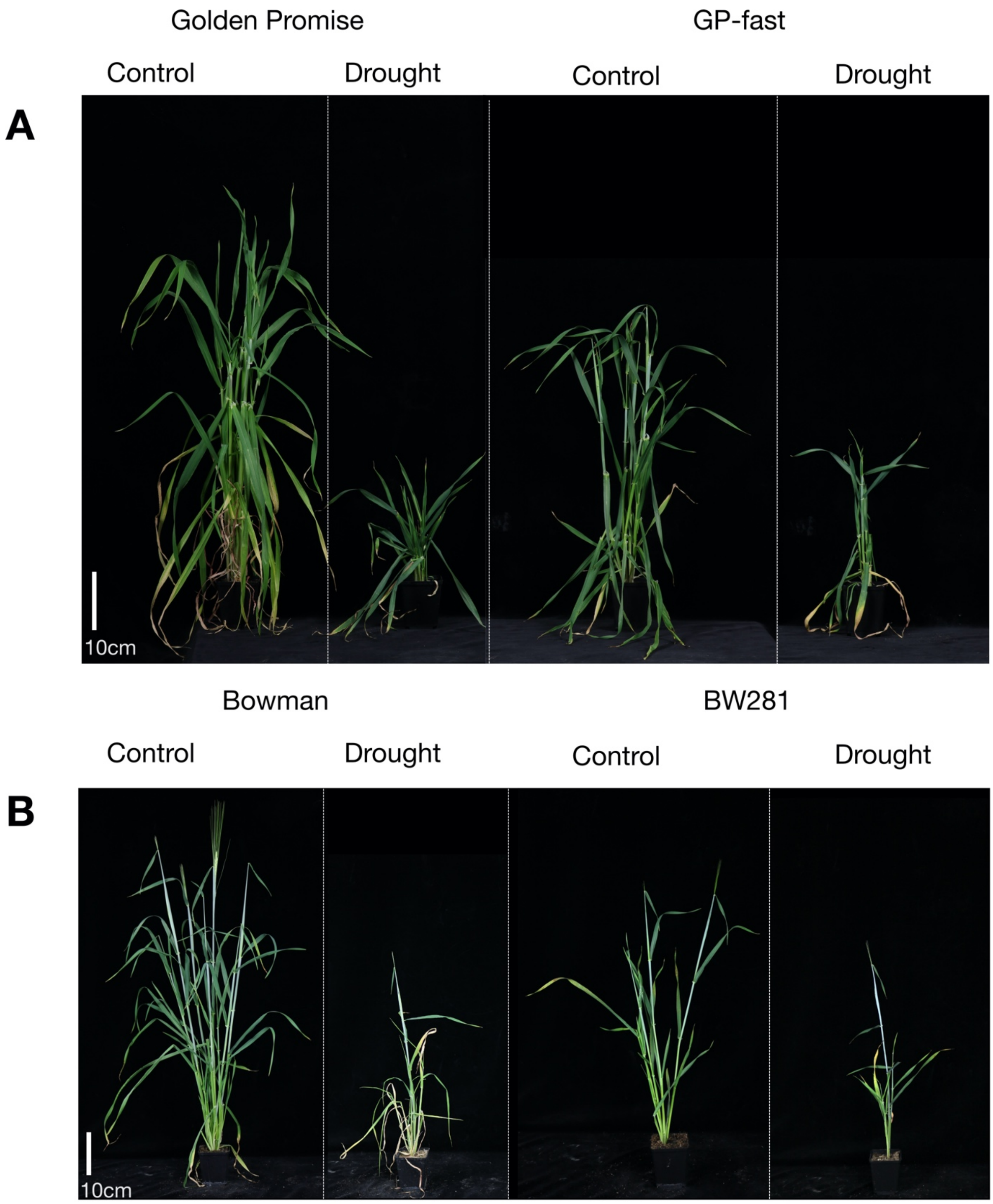

Supplemental Figure 2 Flowering morphology in Golden Promise and Bowman background under continuous drought. Representative plants of Golden Promise and GP-fast (A) plants at heading under control conditions after 63 and 43 DAE respectively. Representative plants of Bowman and BW281 (B) at heading under control conditions after 43 and 35 DAE respectively. Plants were grown under LDs (16 h light/8 h night) under either drought or control conditions.

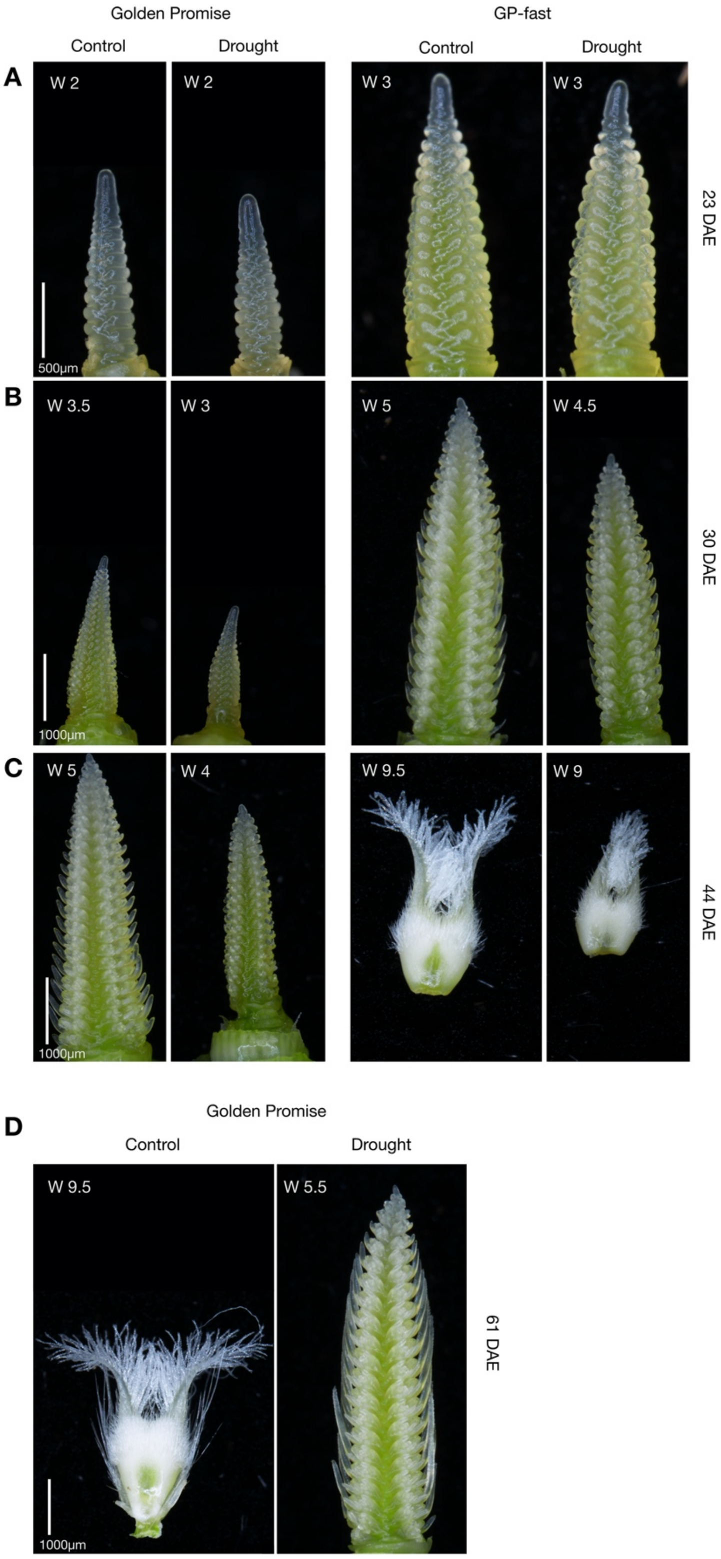

Supplemental Figure 3 MSA and pistil morphology in Golden Promise background. Representative images of MSAs or pistils of Golden Promise and GP-fast 23 DAE (A), 30 DAE (B), 44 DAE (C) and 62 DAE (D). No images of GP-fast are presented for 62 DAE because plants had already flowered. Plants were grown under LDs (16 h light/8 h night) under either drought or control conditions.

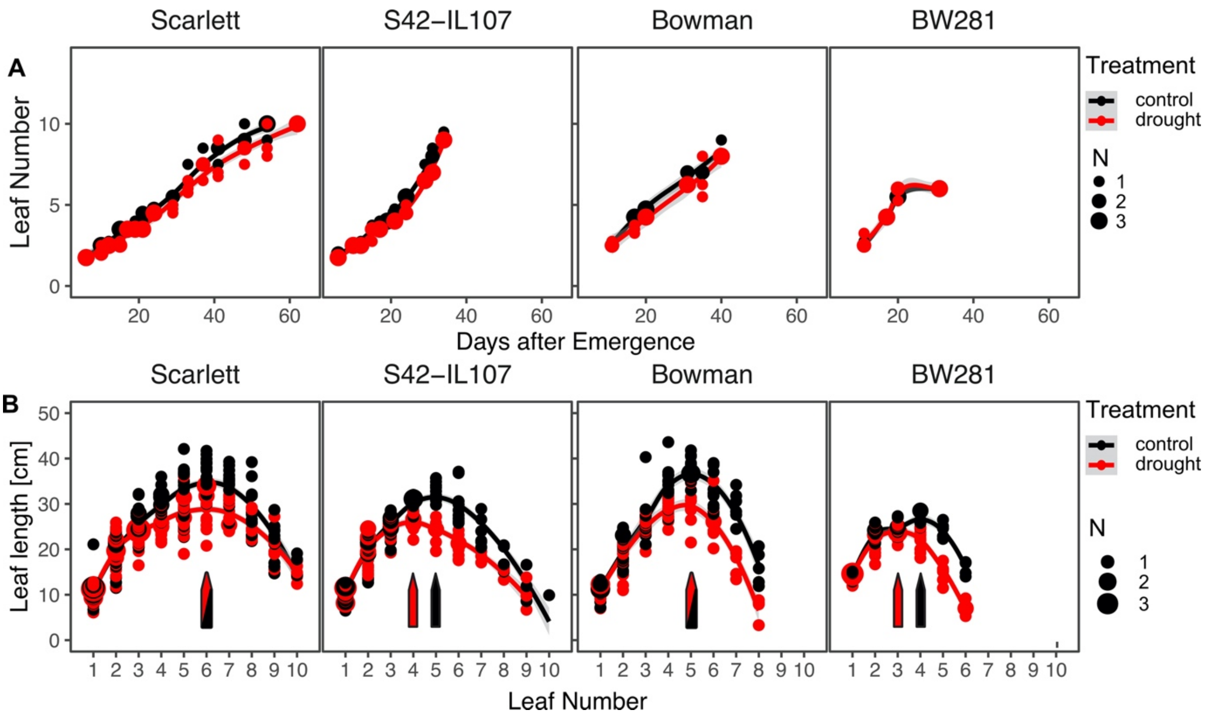

Supplemental Figure 4 Continuous drought affects leaf size but not the phyllochron in barley. Leaf emergence (A) and length of fully emerged leaves (B) were scored over development under control (black) and drought (red) conditions in LDs (16 h light/8 h night) in the spring barley cultivars Scarlett and Bowman and their derived introgression lines S42-IL107 and BW281. Dot sizes indicate the number of overlapping samples. Trend lines were calculated using a polynomial regression (Loess smooth line), grey areas show 95% confidence interval.

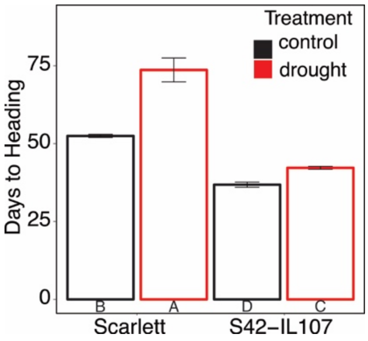

Supplemental Figure 5 Severe drought delays flowering in barley. Days to heading was scored under control (black) and drought (red) conditions in LDs (16 h light/8 h night) in the spring barley cultivar Scarlett and the derived introgression line S42-IL107. Statistical groups were assigned using Kruskal-Wallis ANOVA and post-hoc Conover-Iman-test and Bonferroni correction. Different letters indicate groups differ ($p < 0.05$).
